# Suspected heroin-related overdoses incidents in Cincinnati, Ohio: A spatiotemporal analysis

**DOI:** 10.1371/journal.pmed.1002956

**Published:** 2019-11-12

**Authors:** Zehang Richard Li, Evaline Xie, Forrest W. Crawford, Joshua L. Warren, Kathryn McConnell, J. Tyler Copple, Tyler Johnson, Gregg S. Gonsalves

**Affiliations:** 1 Department of Biostatistics, Yale School of Public Health, New Haven, Connecticut, United States of America; 2 Yale College, New Haven, Connecticut, United States of America; 3 Department of Ecology and Evolutionary Biology, Yale University, New Haven, Connecticut, United States of America; 4 Department of Statistics & Data Science, Yale University, New Haven, Connecticut, United States of America; 5 Yale School of Management, New Haven, Connecticut, United States of America; 6 Yale School of Forestry & Environmental Studies, New Haven, Connecticut, United States of America; 7 Department of Epidemiology of Microbial Diseases, Yale School of Public Health, New Haven, Connecticut, United States of America; 8 Yale Law School, New Haven, Connecticut, United States of America; Massachusetts General Hospital, UNITED STATES

## Abstract

**Background:**

Opioid misuse and deaths are increasing in the United States. In 2017, Ohio had the second highest overdose rates in the US, with the city of Cincinnati experiencing a 50% rise in opioid overdoses since 2015. Understanding the temporal and geographic variation in overdose emergencies may help guide public policy responses to the opioid epidemic.

**Methods and findings:**

We used a publicly available data set of suspected heroin-related emergency calls (*n* = 6,246) to map overdose incidents to 280 census block groups in Cincinnati between August 1, 2015, and January 30, 2019. We used a Bayesian space-time Poisson regression model to examine the relationship between demographic and environmental characteristics and the number of calls within block groups. Higher numbers of heroin-related incidents were found to be associated with features of the built environment, including the proportion of parks (relative risk [RR] = 2.233; 95% credible interval [CI]: [1.075–4.643]), commercial (RR = 13.200; 95% CI: [4.584–38.169]), manufacturing (RR = 4.775; 95% CI: [1.958–11.683]), and downtown development zones (RR = 11.362; 95% CI: [3.796–34.015]). The number of suspected heroin-related emergency calls was also positively associated with the proportion of male population, the population aged 35–49 years, and distance to pharmacies and was negatively associated with the proportion aged 18–24 years, the proportion of the population with a bachelor's degree or higher, median household income, the number of fast food restaurants, distance to hospitals, and distance to opioid treatment programs. Significant spatial and temporal heterogeneity in the risks of incidents remained after adjusting for covariates. Limitations of this study include lack of information about the nature of incidents after dispatch, which may differ from the initial classification of being related to heroin, and lack of information on local policy changes and interventions.

**Conclusions:**

We identified areas with high numbers of reported heroin-related incidents and features of the built environment and demographic characteristics that are associated with these events in the city of Cincinnati. Publicly available information about opiate overdoses, combined with data on spatiotemporal risk factors, may help municipalities plan, implement, and target harm-reduction measures. In the US, more work is necessary to improve data availability in other cities and states and the compatibility of data from different sources in order to adequately measure and monitor the risk of overdose and inform health policies.

## Abstract 2

Gregg Gonsalves and colleagues study associations of features such as the built environment and demographic characteristics with heroin-related overdoses in a US city.

## Author summary

**Why was this study done?:**

- Opioid-related overdose has become one of the most common causes of death in the United States.
- Information about the spatiotemporal pattern of overdose emergencies may be useful in guiding policy responses to the opioid crisis.

**What did the researchers do and find?:**

- We used publicly available data on emergency medical calls to map incidents of suspected heroin-related incidents to census block groups in Cincinnati between August 1, 2015, and January 30, 2019.
- We developed a spatiotemporal statistical model that assessed the association between the number of suspected overdose incidents and demographic, socioeconomic, and environmental characteristics in census block groups.
- We identified features of the built environment and demographic characteristics associated with a higher numbers of heroin-related calls in areas of the city of Cincinnati.

**What do these findings mean?:**

- Understanding the spatiotemporal risk of suspected heroin-related overdose and block group characteristics could inform the design and targeting of public health interventions in the future.
- Greater access to real-time emergency medical services data would help researchers and policymakers better understand the risk of suspected heroin-related overdose.

## Introduction

Drug overdoses claimed over 70,000 lives in the United States in 2017 [1], representing the leading cause of death among Americans under 50 years old [2]. Two-thirds of the drug overdose deaths involved opioids, including heroin, fentanyl, fentanyl analogs, morphine, codeine, hydrocodone, and oxycodone [1]. In 2016, opioid overdoses represented 1 in 65 deaths in the US and 1 in 5 deaths among adults aged 25–34 years [3]. The years of life lost from opioid-related deaths is greater than those associated with hypertension, HIV and/or AIDS, and pneumonia [3]. In both 2016 and 2017, the state of Ohio had the second highest rate of overdose deaths in the country [4]. Hamilton County, which includes Cincinnati and its close suburbs, reported 240 opioid-related fatal overdoses (414 total overdoses) in 2015, rising over 50% to 373 opioid-related fatal overdoses (529 total overdoses) in 2017 [5].

To date, most research on the trends in opioid-related events such as overdose incidents, prescription opioid dispensation, and mortality has focused on the disparities between demographic groups [6, 7] or the spatial disparities among larger geographic areas, at the level of counties [8–10] or of local government areas (LGAs) [11]. Small area analysis of overdose mortality has been carried out mostly using hospital data [8, 12, 13] or data from medical examiner records [14]. Such data, however, are usually subject to delays in reporting and sometimes misclassification of causes of death [15, 16]. In order to effectively deploy policies and strategies for prevention, it is important to understand the spatial and temporal distributions of overdose risk in a timely manner. For example, naloxone is effective at reversing suspected opioid overdose [17], and early administration of the drug is critical for preventing fatal events. Thus, it is important to identify areas with high risks for deploying medical service teams or locating publicly available naloxone kits. Other interventions for people who use drugs, including outreach programs for opioid agonist treatment programs and syringe exchange to prevent HIV transmission could also be more efficiently deployed with better data on the timing and location of overdoses [18].

In most cases, states and emergency medical service (EMS) agencies have time limits within which patient care records must be submitted (24–72 hours), offering more timely information about suspected overdoses. EMS dispatch datasets usually also have high spatial resolution, with global positioning system (GPS) locations or addresses in the call records, making them a valuable resource for understanding when and where each overdose incident happens [19] and for developing opioid use harm-reduction programs [20]. Recently, Carter and colleagues [21] compared spatial concentration of EMS calls, opioid overdose deaths, and crimes in Marion County, Indiana, in order to guide police interventions. Dworkis and colleagues [22] studied the spatial clustering of 700 EMS calls in Cambridge, Massachusetts, that involved opioid overdose to identify clusters amenable to publicly deployed naloxone sites. Dodson and colleagues [23] conducted a similar analysis in Pittsburgh, Pennsylvania, to target pharmacies for naloxone distribution. EMS calls labeled by the dispatcher as related to overdose or opioids may not represent all such incidents, and calls to EMS may be incorrectly labeled by dispatchers as heroin-related based on information obtained from the caller. Despite potential incompleteness, information from EMS calls is often the most timely and readily available data to local governments for real-time response [24, 25]. Currently, counties in 48 of 50 US states already use EMS data as part of the federal Overdose Detection Mapping Application Program (ODMAP) system, relied on by law enforcement, public health, and public safety officials around the country to guide response to overdose [26]. Statistical analysis of the publicly available EMS data, combined with information on demographic and neighborhood characteristics, could further provide a better quantitative and qualitative understanding of local overdose risk for public health experts and frontline service providers.

In this paper, we present a spatiotemporal analysis of the locations of reported heroin-related incidents associated with EMS dispatches in the city of Cincinnati, Ohio. We investigated the spatial and temporal variability as a function of economic and demographic covariates, accessibility of medical facilities, and features of the built environment. We employed a hierarchical Bayesian discrete space-time regression model at the census block group level to account for spatial and temporal correlation that cannot be explained by geographic and demographic covariates.

## Materials and methods

Additional details describing the data and methods are available in S1 Appendix. The analyses here were not prespecified. Our analytic approach used methods widely employed in spatial epidemiology and selection of covariates was based on their association with overdose or substance use in the literature. This study is reported as per the Strengthening the Reporting of Observational Studies in Epidemiology (STROBE) guidelines [27] in S1 STROBE Checklist.

### Cincinnati EMS data

EMS response data related to heroin overdose were obtained from the City of Cincinnati's computer-aided dispatch (CAD) database [28]. The EMS data are publicly available and capture all responses by the Cincinnati Fire Department to reported heroin overdose incidents. An incident is classified as heroin-related by the dispatcher if the situation is described by the caller as involving heroin or opiates, or the caller describes behavior related to heroin use (e.g., injection using a syringe) [28, 29]. Cincinnati EMS calls labeled as suspected "heroin-related" incidents by dispatchers may include incidents related to overdose or poisoning with other non-heroin or non-opioid substances. For simplicity in what follows, we use the term "heroin-related incidents" when referring to the outcome of interest. Almost all incidents were associated with GPS locations assigned by the Cincinnati Office of Performance and Data Analytics, which anonymizes locational data by randomly offsetting the original coordinate values to within approximately 100 yards in any direction [29].

We extracted all EMS call records labeled as "heroin-related" during the period of August 1, 2015, to January 31, 2019. In order to understand the association between the distribution of heroin-related incidents and spatial covariates, we mapped all incidents to the census block groups of Cincinnati using the GPS locations. A small number of block groups fall partially outside the city boundary or within neighboring jurisdictions. For this analysis, we used 280 block groups with at least 50% area within the city boundary. The discretization into block groups allowed us to relate the number of incidents to demographic and socioeconomics covariates at a high spatial resolution. We also conducted a sensitivity analysis by including both heroin-related incidents and incidents labeled as "overdose/poisoning (ingestion)" as the outcome and summarized the results in S1 Appendix.

### Covariates

**Fig 1:**
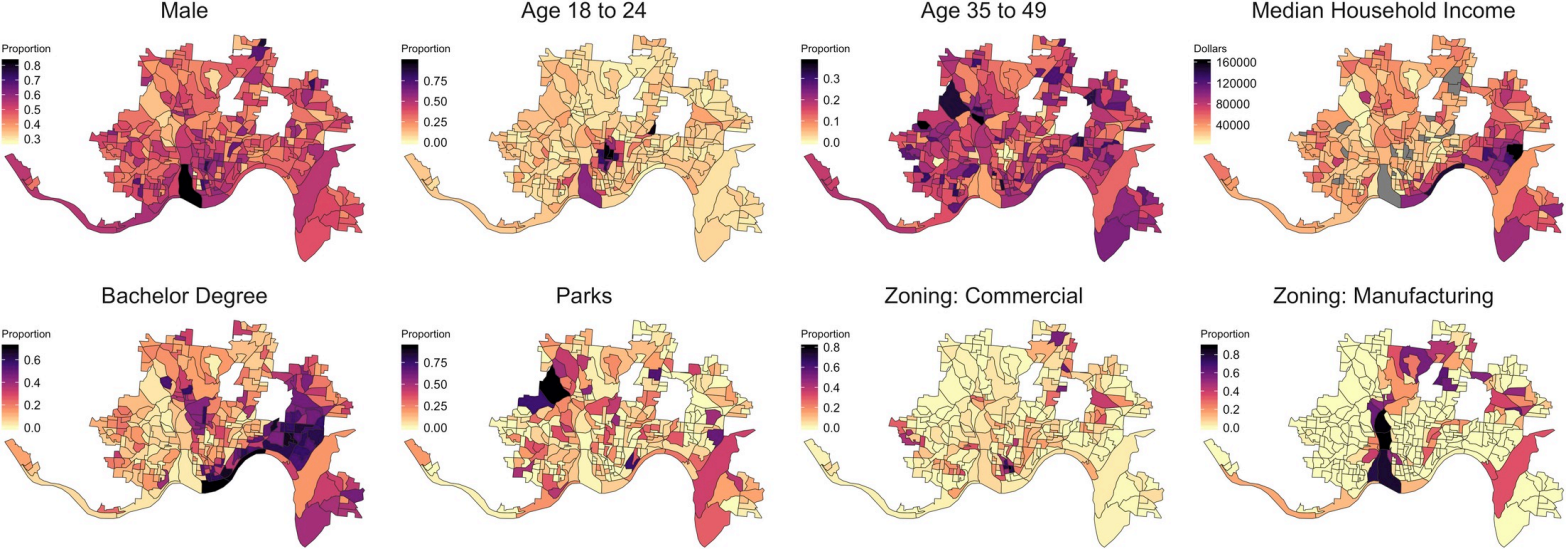
Summary of a subset of time-invariant covariates by block groups. Gray areas indicate that no reliable estimates are available from American Community Survey. Geographic boundary files were downloaded from the US Census, TIGER, Geodatabase [41].

We gathered information on population in each block group from the 2013 to 2017 estimates of the American Community Survey [30]. These covariates include the population size, percentage of the population by gender, age group, race, and education, median household income, per capita income, percentage of households below poverty level, and median home values. We also calculated the change in median home values from the 2009 to 2013 estimates of the American Community Survey. A small number of these covariates are missing in the analysis because of small sample size within block groups. For each block group, we calculated measures of accessibility to health facilities, including minimum distance from the the geographic centers of each block group to hospitals [31], pharmacies [31], federally qualified health centers (FQHCs) [31], buprenorphine prescribing physicians, and Substance Abuse and Mental Health Services Administration (SAMHSA) Opioid Treatment Programs (OTPs) [32]; and the distance to the closest fire department [33]. Environmental variables have also been found to be associated with drug use and overdose [34–36]. In order to characterize block groups by features of their built environment relevant to the risk of overdose, we calculated the proportion of areas covered by quarter-mile buffer areas from bus stops, the proportion of park areas using the publicly available GIS data set from the city of Cincinnati [37], and the number of fast food restaurants from the Cincinnati Bell directory [38]. We also obtained zoning maps of the land development code from the city of Cincinnati [37] and calculated the proportion of areas covered by 9 categories of zoning districts, including single-family housing, multifamily, and institutional residential, office, commercial, urban mixed use, downtown development, manufacturing, riverfront, and planned development. A detailed list of covariates is presented in S1 Appendix. Fig 1 shows the spatial variation of selected key covariates. The complete list of covariates and their distributions can be found in S1 Appendix. We also obtained time-varying covariates including monthly counts of crime incidents per resident in each area from the Cincinnati open data portal [39] and monthly average temperature and total precipitation to account for seasonal variation [40].

### Analytic approach

We model monthly counts of incidents by block groups using a Bayesian space-time model that is widely used in disease mapping and spatial epidemiology [42, 43]. We model the number of incidents *y*_*it*_ in block group *i* during month *t* as independently Poisson distributed, given the mean number of incidents *λ*_*it*_,

$$y_{it}\overset{ind.}{∼}Poisson(\lambda_{it}),$$ (1)

and the logarithm of the average number of incidents is modeled as

$$\log(\lambda_{it})=x_{it}^{T}\beta +\alpha_{i}+\varphi_{t}+\delta_{it},$$ (2)

where ***x***_*it*_ is the vector of covariates for block group *i* at time *t* and *β* is a vector of fixed effect regression parameters. The covariate vector, ***x***_*it*_, includes both time-varying and spatially varying variables described above. The natural logarithm of the rate *λ*_*it*_ was modeled as the sum of effects from covariates and effects of block groups, time, and interaction terms. The random effects approximate the additional variation not explained by the covariates. In this analysis, we treat log population as a covariate; an analysis with population size as a multiplicative offset is presented in S1 Appendix.

The spatial term for the *i*th block group, *α*_*i*_, is a spatially structured random effect that follows the Besag, York, and Mollié (BYM) model [44]. The random effect can be further decomposed into an intrinsic conditional autoregressive term [45] in which the value at a particular location depends on the values at neighboring locations, plus independent location-specific error. Because the EMS call data consist of only locations within Cincinnati, we treat the block groups within the 2 enclaved regions as missing data in our analysis, so that the structure of the surrounding regions are properly accounted for. The temporal trend *φ*_*t*_ is modeled by the sum of 2 terms: an autoregressive model of order 1, which is a random process that depends linearly on its previous value and a stochastic term, and an unstructured independent temporal noise term. Finally, the space-time interaction term *δ*_*it*_ is modeled as an independent noise term for each block and time period to account for local “shocks” that deviate from the average level and trend.

Posterior distributions of parameters and the modeled incident counts ${\hat{y}}_{it}$ conditional on the observed data are obtained by fitting this hierarchical Bayesian model [46] using the integrated nested Laplace approximation method implemented with the R-INLA package [47] in the R statistical programming environment [48]. Additional details on model specification and comparison can be found in S1 Appendix.

### Ethical approval

Public use data sets, such as the EMS data captured by the Cincinnati Fire Department on overdose incidents, are prepared with the intent of making them available for the public. These data are not individually identifiable or maintained in a readily identifiable form. We did not merge any of the data sets in such a way that individuals might be identified and did not enhance the public data set with identifiable or potentially identifiable data. Thus, this work does not constitute human subjects research and does not require ethical approval.

## Results

**Fig 2:**
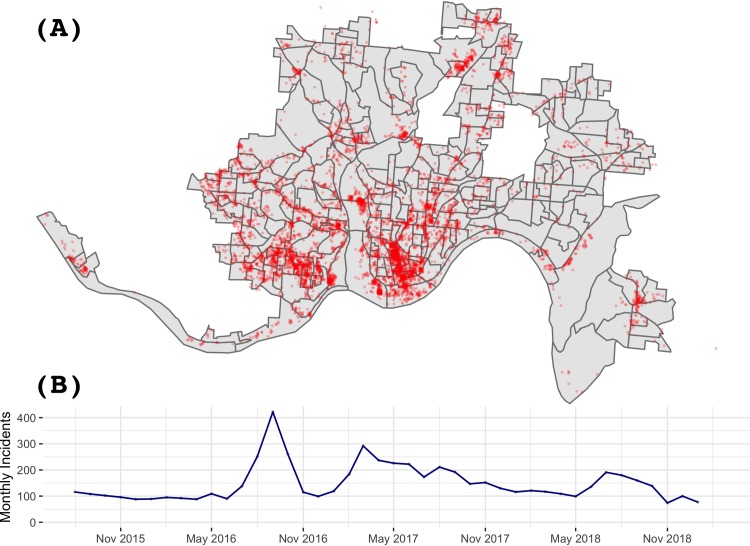
Heroin-related calls to emergency medical services in Cincinnati, Ohio, from August 2015 to January 2019. (A) Locations of incidents over the study. There are 2 enclaved areas in the map, consisting of 3 cities or villages surrounded by the city of Cincinnati (Norwood, St. Bernard, and the village of Elmwood Place). EMS data for these 2 areas are not available. Geographic boundary files were downloaded from the US Census, TIGER, Geodatabase [41]. (B) number of incidents by month. EMS, emergency medical service.

**Fig 3:**
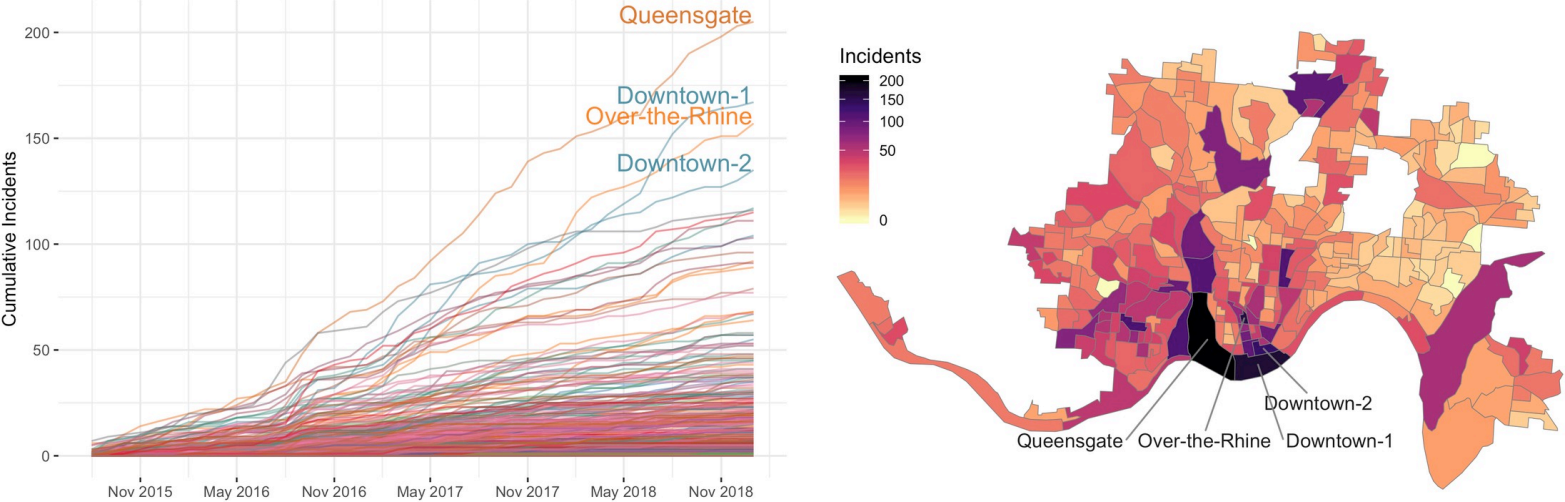
Summary of incidents labeled as heroin-related by block group and month. (Left) Total number of incidents by month. (Right) Total number of incidents by block group. Geographic boundary files were downloaded from the US Census, TIGER, Geodatabase [41].

During the study period, there were a total of 6,264 incidents within the block group boundaries. Fig 2 shows the locations of all heroin-related overdose incidents and the monthly totals. The spatial distribution of incident locations exhibit strong heterogeneity. Several apparent spikes are evident, including 3 major peaks in September 2016, March 2017, and July 2018. The spikes are corroborated by local news reports of rising overdose deaths published around the same time [49–55]. Fig 3 shows the cumulative number of incidents by time and block groups, with the block groups showing the highest number of cumulative incidents indicated on the map.

**Fig 4:**
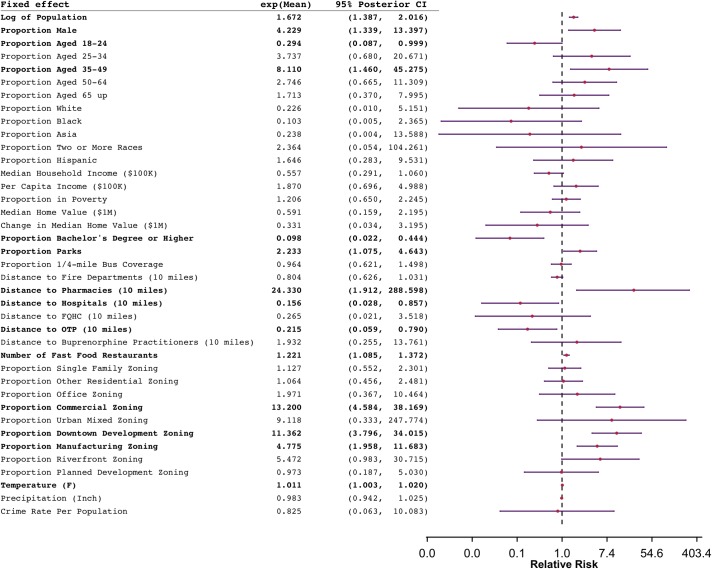
Summary of the posterior means and 95% CIs of the coefficients for the fixed effects. The regression coefficients are exponentiated to represent RRs in the table. CI, credible interval; RR, relative risk.

Posterior distributions of the fixed effects *β* are summarized in Fig 4. Several demographic and socioeconomic covariates of residents are strongly associated with the number of heroin-related incidents (i.e., 95% CIs that exclude 0). The number of heroin-related incidents is positively associated with population size, proportion of male population, and the proportion of residents aged 35 *to* 49, and negatively associated with the proportion of residents aged 18 *to* 24, the proportion of residents with a bachelor's degree or higher, and median household income. Features of the built environment, including the proportion of parks, commercial, manufacturing, and downtown districts and the number of fast food restaurants, exhibit strong positive associations with the number of heroin-related calls. Higher numbers of heroin-related incidents are associated with shorter distances to OTP, shorter distances to hospitals, and longer distances to pharmacies; all 4 OTPs and several hospitals are located within the center of the city where the number of heroin-related incidents is higher. Higher temperature is also found to be positively correlated with the number of heroin-related calls.

**Fig 5:**
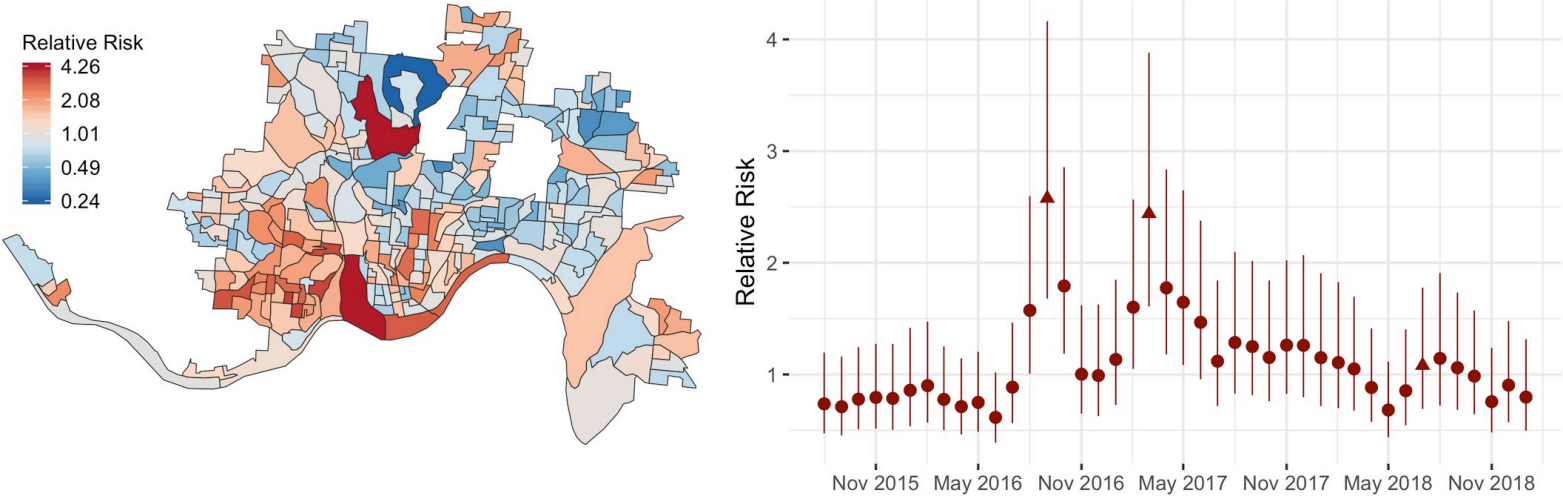
Posterior means of the autoregressive spatial (*α*_*i*_) and temporal (*φ*_*t*_) effects. The random effects are exponentiated to represent RRs. Larger values correspond to higher log counts of incidents. The error bars indicate 95% posterior CIs. The triangle dots correspond to the 3 major peaks in heroin-related incidents at September 2016, March 2017, and July 2018. Geographic boundary files were downloaded from the US Census, TIGER, Geodatabase [41]. CI, credible interval; RR, relative risk.

**Fig 6:**
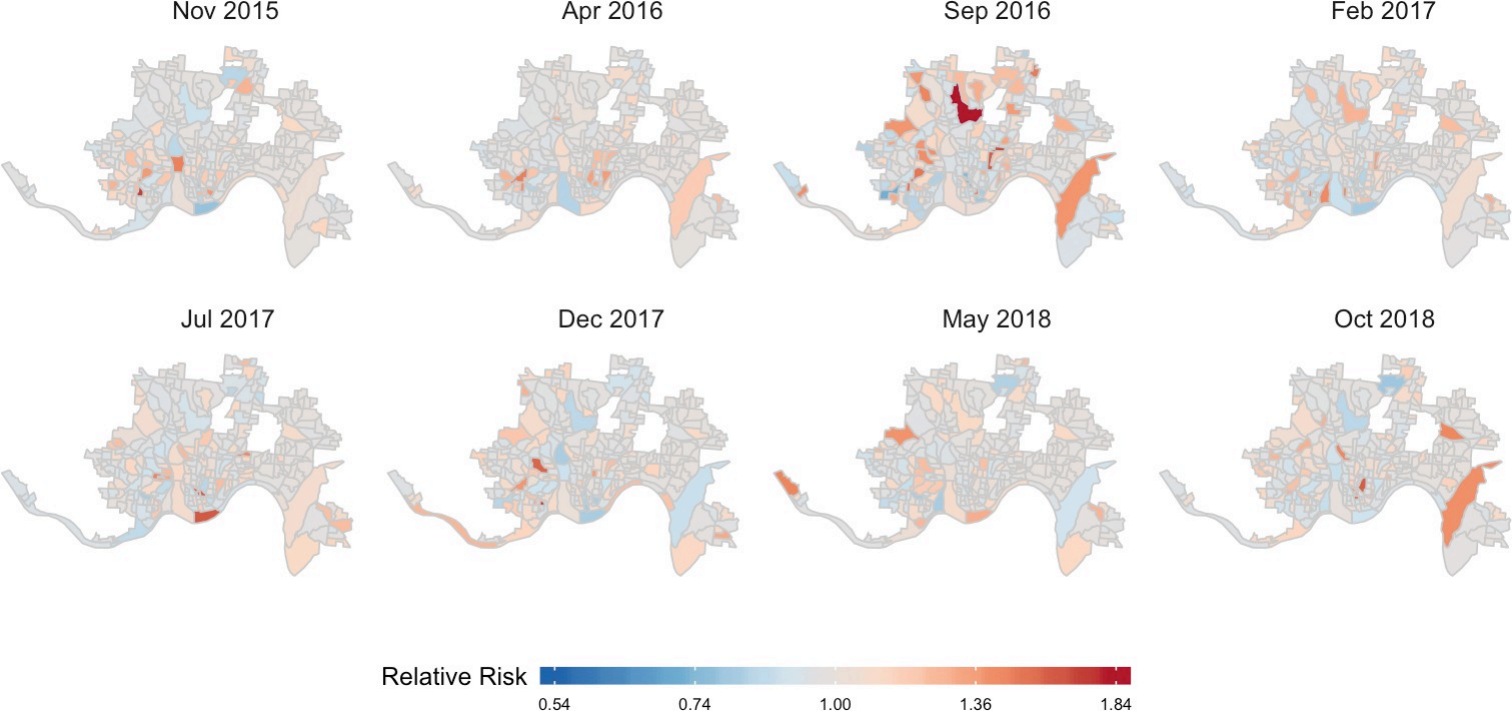
Posterior means of the independent space-time interaction effects (*δ*_*it*_) for a subset of the time periods. The random effects are exponentiated to represent RRs. The areas in red have higher risks of heroin-related incidents than the structured trends over space and time. Geographic boundary files were downloaded from the US Census, TIGER, Geodatabase [41]. RR, relative risk.

**Fig 7:**
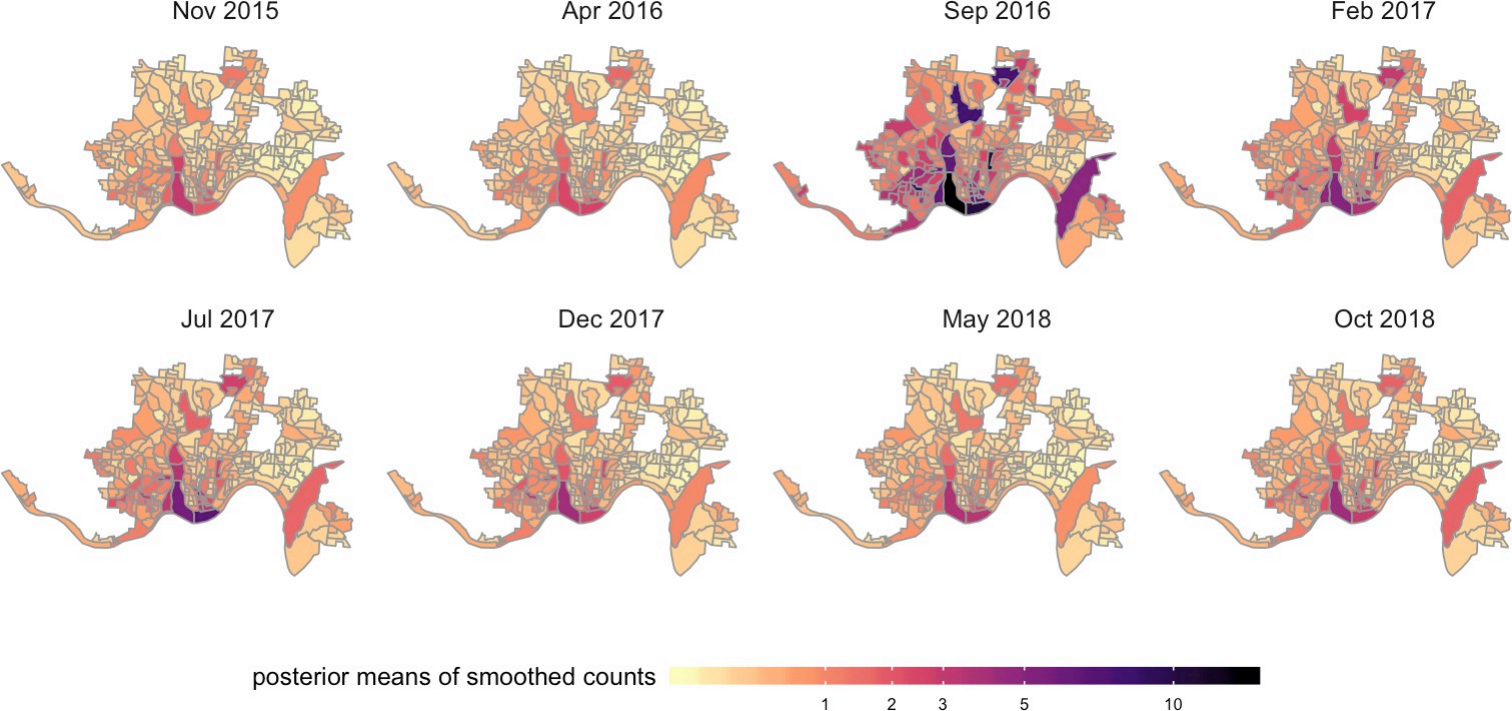
Posterior means of the fitted heroin-related incident counts (${\hat{y}}_{it}$) for a subset of the time periods. Geographic boundary files were downloaded from the US Census, TIGER, Geodatabase [41].

Marginal posterior distributions for the spatial and temporal effects are summarized in Fig 5. The posterior means of the spatial random effects are highly structured with positive values in the southwest part of the city and negative values on the east side. The strong spatial heterogeneity in the posterior of the spatial random effect suggests that there may be spatial variables beyond those included in this analysis that contribute to the imbalance in the number of heroin-related incidents between the east and west side of Cincinnati. The temporal random effects capture the main trends in the number of heroin-related incidents over time. We again see the 3 peaks in heroin-related incidents as shown in Fig 2. The unstructured space-time random effects capture the local deviations from the trends for each area and time period. Fig 6 shows the posterior means of these shocks that are local in space and time, which may be visually assessed to help identify residual hot spots. The modeled incident counts ${\hat{y}}_{it}$ are summarized in Fig 7. The regions with darker colors are associated with higher risks. In addition, compared with the first surge in heroin-related incidents, the later 2 correspond to areas with elevated risks that are spatially more clustered in the west and southwest parts of the city. Additional results under different model and data specifications can be found in S1 Appendix.

## Discussion

In this analysis, we identified areas with a high number of calls labeled as heroin-related incidents by EMS dispatchers in the city of Cincinnati, along with sociodemographic variables and features of the built environment associated with these counts. We used a Bayesian spatiotemporal analysis to associate the reported number of heroin-related incidents to covariates at the level of census block groups. We identified significant associations between the number of heroin-related calls and demographic characteristics of residents and features of the built environment. We found that spatial and temporal heterogeneity remain after adjusting for all measured covariates.

This study has a few limitations. First, we studied overdose incidents classified as heroin-related at the time of dispatch. The conclusion on the scene may be different but recoding does not occur on site. In addition, more general classifications for dispatches, for instance, coded as overdose or person down, may also be heroin-related. Though we evaluated the sensitivity to potential misclassification and found no significant bias, more efforts in linking data sets collected from different sources could potentially provide more complete counts of heroin-related overdose incidents. Second, the spatiotemporal analysis conducted is ecological in nature and cannot be interpreted as characterizing individual-level risk factors for overdose. In addition, people affected by overdose in a given geographic area may not actually reside there. However, the results of such studies may guide localized interventions that target small areas with high overdose incident frequency. Third, a major source of temporal variation may be changes in local policy, enforcement, and intervention responses to the increasing rates of overdose. Fourth, although the method can be used for short-term prediction of heroin-related overdose activities, the model may not provide reliable predictions for when and where the sharp increase in overdoses will happen with the available data. More timely dissemination of information on drug toxicology from hospital and police seizure data may help predict such spikes. However, postmortem drug screening and large-scale seizures of drug shipments may not reflect the complex dynamics of street-level drug supply. Although being able to predict transient increases in overdoses may be important, predicting where overdoses are most likely to happen over time may offer ways to prevent these increases in the first place and may be the more critical public health task. For instance, with widespread dissemination of fentanyl test strips, in areas of high risk of overdose, people who inject drugs (PWID) could determine the composition of their drug supply, which may guide safer use. Finally, the classification of calls as heroin-related may not capture overdose incidents related to nonmedical prescription of opioids [56], which are estimated to contribute from a quarter to a half of overdoses in the US in 2016 [57].

Understanding the exogenous factors that might spur increased overdose activities in the city will take further analysis. Several spikes in overdoses apparent in the EMS calls have also been reported in both local and national news. Immediately after the first uptick in late August 2016, it was reported on September 2nd that Cincinnati officials had obtained more naloxone kits, planned to push for funding for community-based opioid overdose recognition and response training programs, and created a quick response team to revisit overdose victims within 2 weeks [51]. Evidence of carfentanil-laced heroin on the market during this period subsequently emerged [52]. Compounds of heroin, fentanyl, and carfentanil were also found to be related to overdose deaths in Cincinnati in 2017 during the second spike [58]. However, following the later 2 major surges of overdose, the number of overdose incidents decreased at a much slower pace. A closer examination of the differences among the 3 periods of increased overdose incidents in terms of both the source of surge and the actions taken in response may reveal useful lessons in understanding and fighting the epidemic.

This analysis provides inferences based on the current state, scope, and availability of data on heroin-related EMS calls in Cincinnati. EMS data, as well as data from other first responders, and additional demographic, social, and economic covariates derived from local knowledge of community characteristics may be helpful in further refining responses to the opioid crisis. To generalize this analysis to other locations will require selection of covariates based on local knowledge, because the use of opioids and the social, economic, and political context of this use may differ between urban and rural areas [59] and from country to country [60]. However, obtaining this information in other locations can be difficult, even though some of it (e.g., EMS call data) is collected in near real time in most US states via the National EMS Information System [61]. Our attempts to obtain geocoded, time-stamped EMS data from several states and municipalities have been unsuccessful, even under data use and privacy agreements. This analysis was conducted using data that were available for public use and scrutiny, but such open data policies are not widely shared by other jurisdictions in the US. Cincinnati has made the data used for this study available as part of its greater Open Data Cincinnati initiative [62], and other databases that integrate fire and police calls every 15 minutes are maintained by the city's Office of Performance and Data Analytics [33].

Greater access to near-real-time data sources will be important in deepening our understanding of the spatiotemporal aspects of overdose. In addition, linking related sources of data, for instance, EMS data with data on fatal overdoses from medical examiner and coroner's offices, with naloxone administration by first responders other than EMS personnel (e.g., police, bystanders) in a standardized format would enrich these kinds of analyses and make their execution easier. The data used in this analysis are primarily operational and administrative records and were not collected for the purpose of overdose surveillance. However, states like Massachusetts, through Chapter 55 legislation, have begun to link multiple databases to better understand the opioid epidemics in their jurisdictions, with the explicit goal of allowing cooperative data analysis with research partners [63]. Making this information more widely available on a timely basis, linking databases, standardizing formats, and building systems to allow for analysis such as those performed here, will be vital to inform public health policy and practice to address the overdose epidemic.

## Supporting information

S1 STROBE Checklist The STROBE checklist of items that should be included in report. STROBE, Strengthening the Reporting of Observational Studies in Epidemiology
(DOC)

S1 Appendix The appendix contains a detailed presentation of the data, methods, and additional model validation results. (PDF)

## Abbreviations

CAD: computer-aided dispatch
CI: credible interval
EMS: emergency medical service
FQHC: federally qualified health center
GPS: global positioning system
LGA: local government area
ODMAP: Overdose Detection Mapping Application Program
OTP: Opioid Treatment Program
PWID: people who inject drugs
RR: relative risk
SAMHSA: Substance Abuse and Mental Health Services Administration
STROBE: Strengthening the Reporting of Observational Studies in Epidemiology
