# Supplementary material for: Suspected heroin-related overdoses incidents in Cincinnati, Ohio: A spatiotemporal analysis
Source: PLoS Med. 2019 Nov 12;16(11):e1002956. doi: 10.1371/journal.pmed.1002956 (PMC6850525; doi:10.1371/journal.pmed.1002956)
Supplement: S1 Appendix — (PDF) [file pmed.1002956.s002.pdf]

Supplementary Materials to *Suspected heroin-related overdose incidents in Cincinnati, Ohio: a spatiotemporal analysis*

Zehang Richard Li[1], Evaline Xie[2], Forrest W. Crawford[1,3,4,5], Joshua L. Warren[1], Kathryn McConnell[6], J. Tyler Copple[7], Tyler Johnson[7], and Gregg S. Gonsalves[7,8]

1. Department of Biostatistics, Yale School of Public Health
2. Yale College
3. Department of Ecology and Evolutionary Biology, Yale University
4. Yale School of Management
5. Department of Statistics & Data Science, Yale University
6. Yale School of Forestry & Environmental Studies
7. Department of Epidemiology of Microbial Diseases, Yale School of Public Health
8. Yale Law School

September 23, 2019

Contents

1 EMS data **2**

1.1 List of removed EMS disposition codes 2

1.2 Map of Cincinnati and block group selection 2

1.3 Distribution of EMS calls by type 2

1.4 Spatial-temporal dynamics of overdose incidents 3

2 Spatial and temporal covariates **10**

3 Details of the Space-time Smoothing Model **12**

3.1 Hyperprior choices 12

3.2 Posterior distribution of hyperparameters 12

3.3 Model selection 12

3.4 Predictive performance . . . 14

3.5 Sensitivity to jittered GPS location . . . 14

3.6 Sensitivity to incident type classification . . . 19

3.7 Seasonality . . . 26

1 EMS data

1.1 List of removed EMS disposition codes

Events in the EMS dataset that correspond to duplicated, test, canceled events, false alarms, and events that no units are dispatched, or the dispatched unit was disregarded before arriving, are removed from the analysis. The following list consists of the disposition codes that are removed: cancel, nothing to report, duplicate, test, not a disposition, no violation, false alarm, error incident, advised, good intent, reassigned , EMSF: false medical situation, MEDF: medical response, false, FD: fire disregard, EMSD: medics disregard, MEDD: medics transport disregarde, and EXT: extinguish.

1.2 Map of Cincinnati and block group selection

Figure 1 shows the neighborhood map of Cincinnati. The two major enclaved areas within Cincinnati, the City of Norwood, and the City of St Bernard, and the Village of Elmwood Place, are indicated on the map. Figure 2 shows the structure of block groups in the enclaved areas. EMS data for these areas are not available and are treated as missing values, and imputed, in the analysis. Block groups with at least 50% of area within the city of Cincinnati are used in the analysis presented in the main text. Figure 3 illustrates different criterion to select the block groups in the analysis. The resulted block groups maps remain mostly consistent when different thresholds are used.

We fit the model for each of the nine different thresholds shown in Figure 3. Figure 4 compares the estimated fixed effects in the main analysis to the parameters estimated from these nine experiments. Figure 5 shows the estimated spatial random effects under the same comparison. Variations in the fixed effects and spatial random effects due to different threshold of block group inclusion are very small and show no significant changes to the conclusions in the main analysis. Figure 6 shows the estimated temporal random effects, which also do not change when the inclusion threshold vary.

1.3 Distribution of EMS calls by type

Figure 7 shows all incident type groups with more than $1,000$ incidents during the study period. No other types of incidents exhibit similar patterns of spikes as heroin-related overdose (solid black line). We also fit the model with incident counts that include those classified as general overdose/poisoning (dotted black line) and obtained similar results.

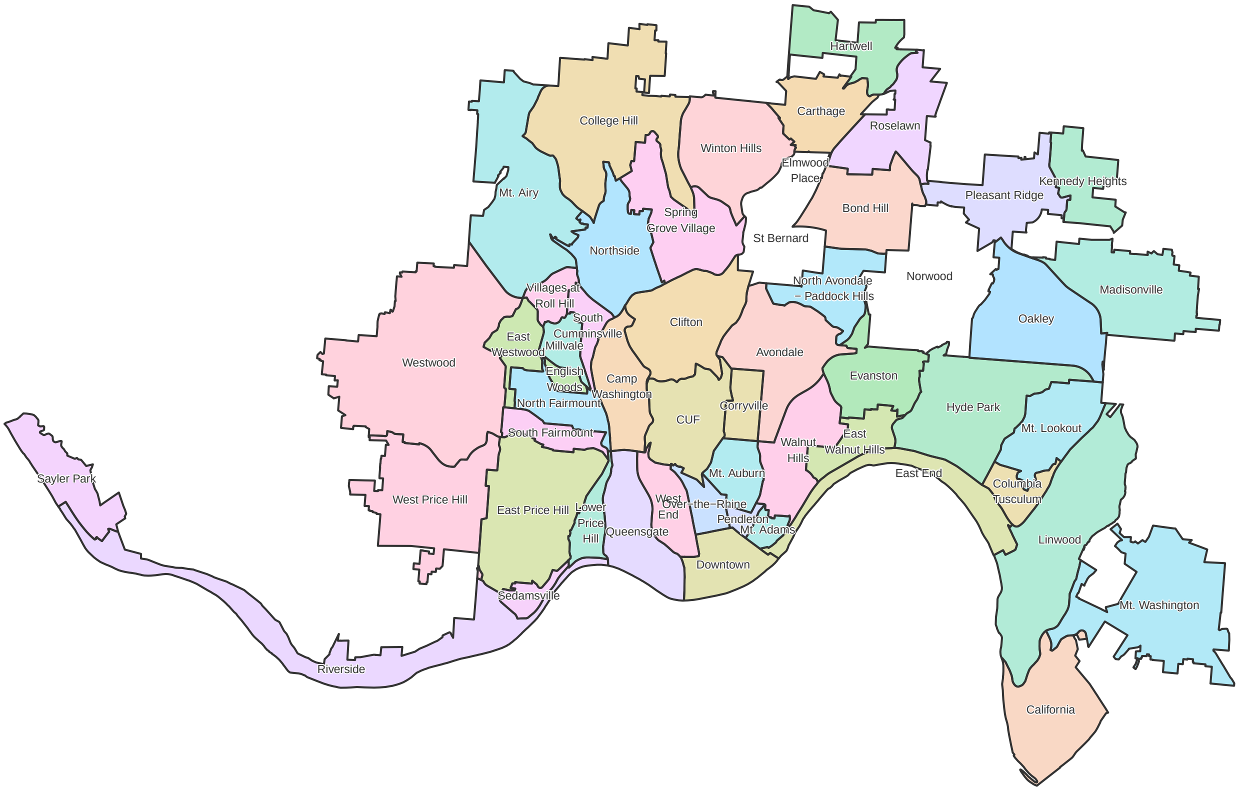

Figure 1: Neighborhood map of Cincinnati, with enclaved regions City of Norwood, the City of St Bernard, and the Village of Elmwood Place, indicated. Geographic boundary files were downloaded from the United States Census, TIGER, Geodatabase (City of Cincinnati, 2010).

1.4 Spatial-temporal dynamics of overdose incidents

Figure 8 shows the counts by block groups respectively in 6-month windows, and also highlights the concentration and dynamic changes of incident rates in small areas within neighborhoods.

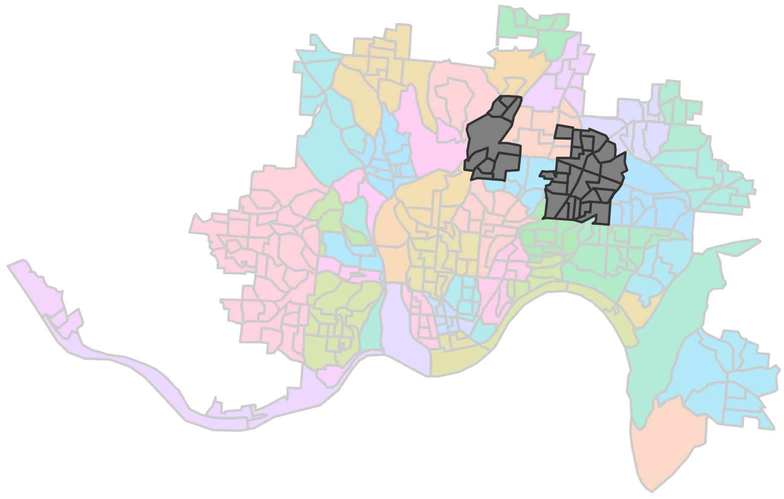

Figure 2: Excluded block groups for the two enclaved areas in Cincinnati. Color indicates the neighborhoods of Cincinnati. Geographic boundary files were downloaded from the United States Census, TIGER, Geodatabase (United States Census Bureau, 2017).

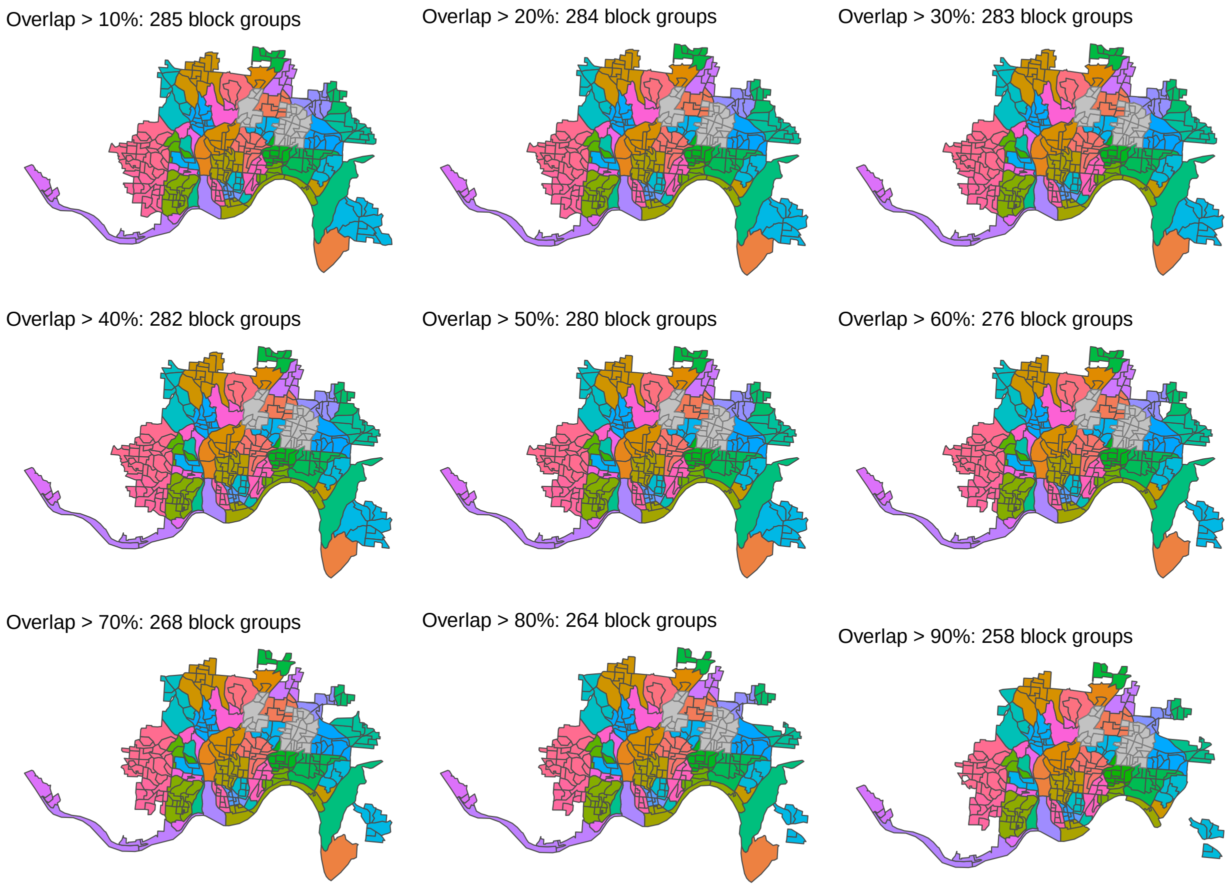

Figure 3: Comparison of different criteria for selection of block groups in the statistical analysis. The maps show the geography of the census block groups with greater than $a\%$ of area within the city of Cincinnati, where $a\%$ varies between 10% and 90%. Color indicates Cincinnati neighborhood. Geographic boundary files were downloaded from the United States Census, TIGER, Geodatabase (United States Census Bureau, 2017).

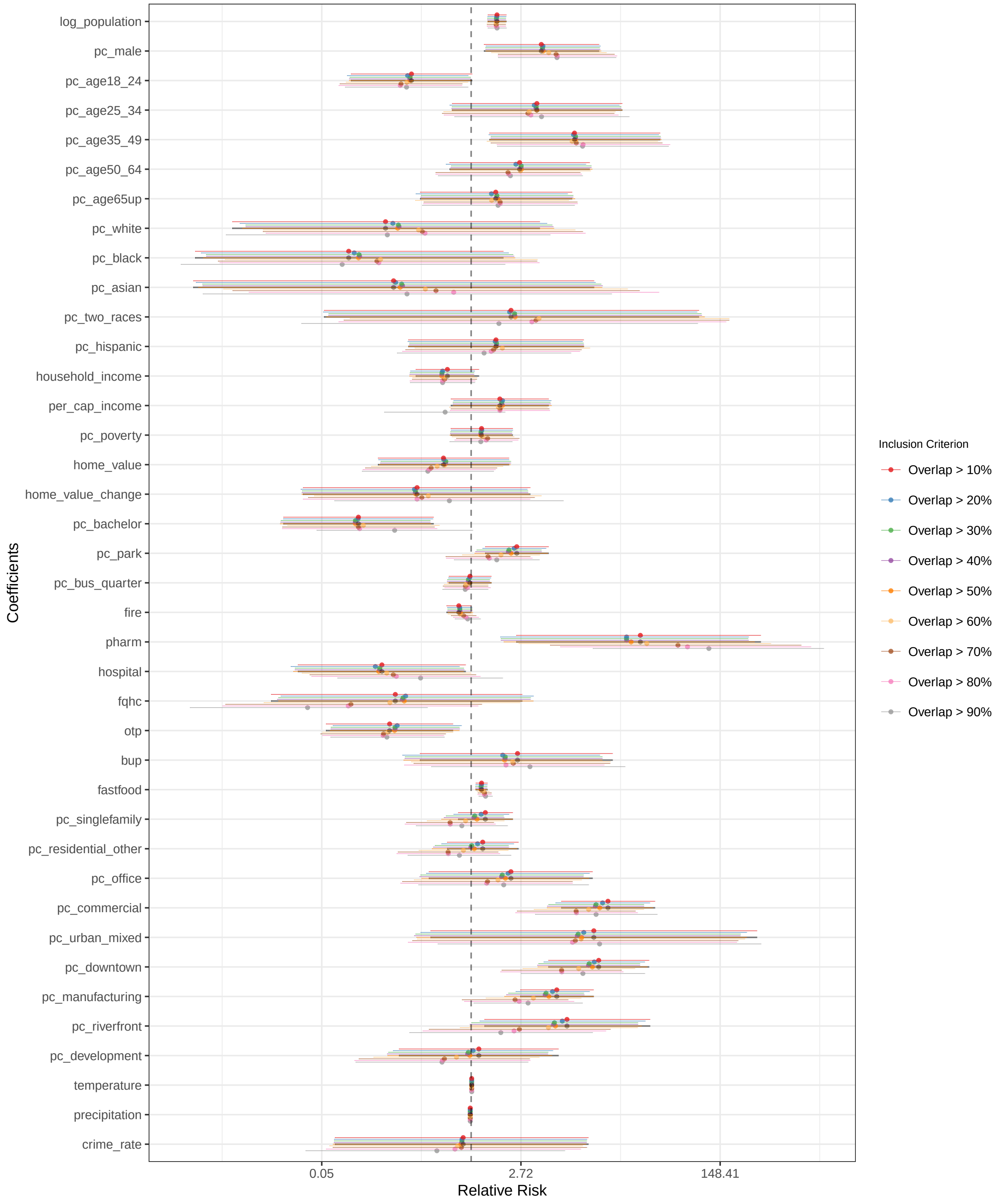

Figure 4: Comparison of posterior means and 95% credible intervals of the fixed effects in the main results (Overlap > 50%) with the posterior means of the fixed effects under varying inclusion criterion of block groups.

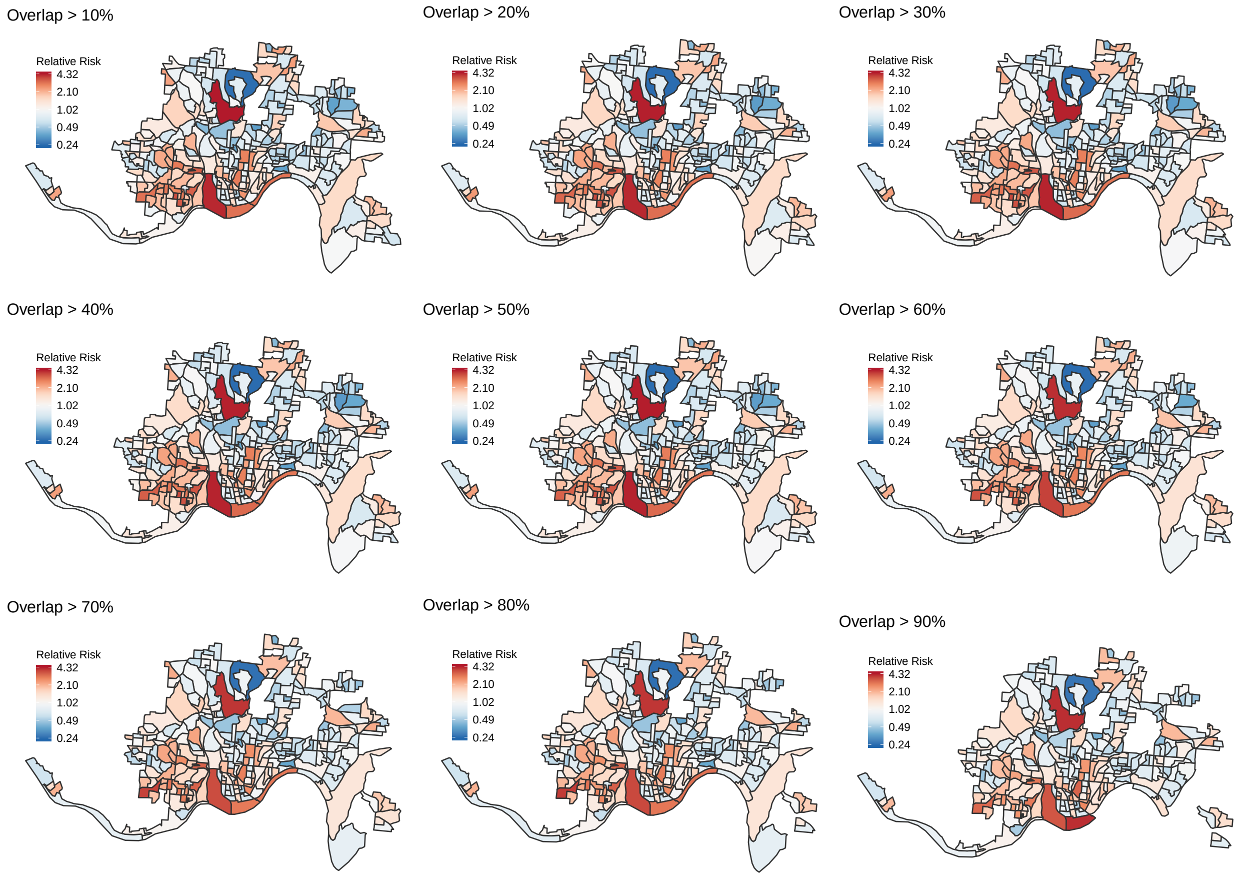

Figure 5: Comparison of posterior means of the spatial random effects in the main results (Overlap > 50%) with the posterior means of the spatial random effects under varying inclusion criterion of block groups. Geographic boundary files were downloaded from the United States Census, TIGER, Geodatabase (United States Census Bureau, 2017).

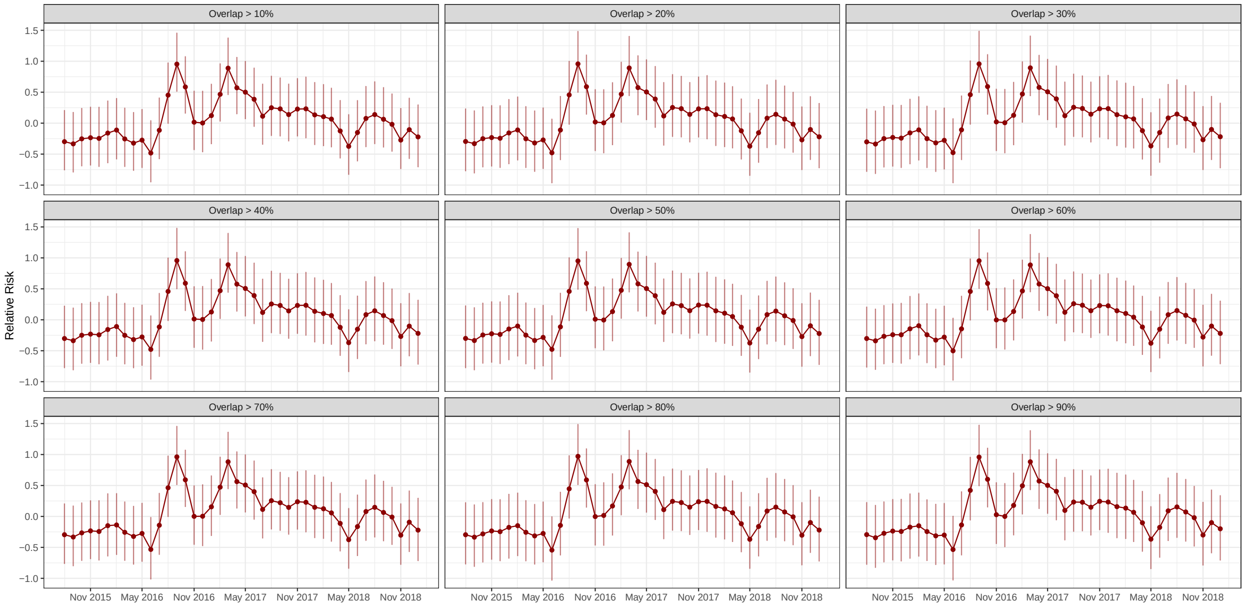

Figure 6: Comparison of posterior means and 95% credible intervals of the temporal random effects in the main results (Overlap > 50%) with the posterior means of the temporal random effects under varying inclusion criterion of block groups.

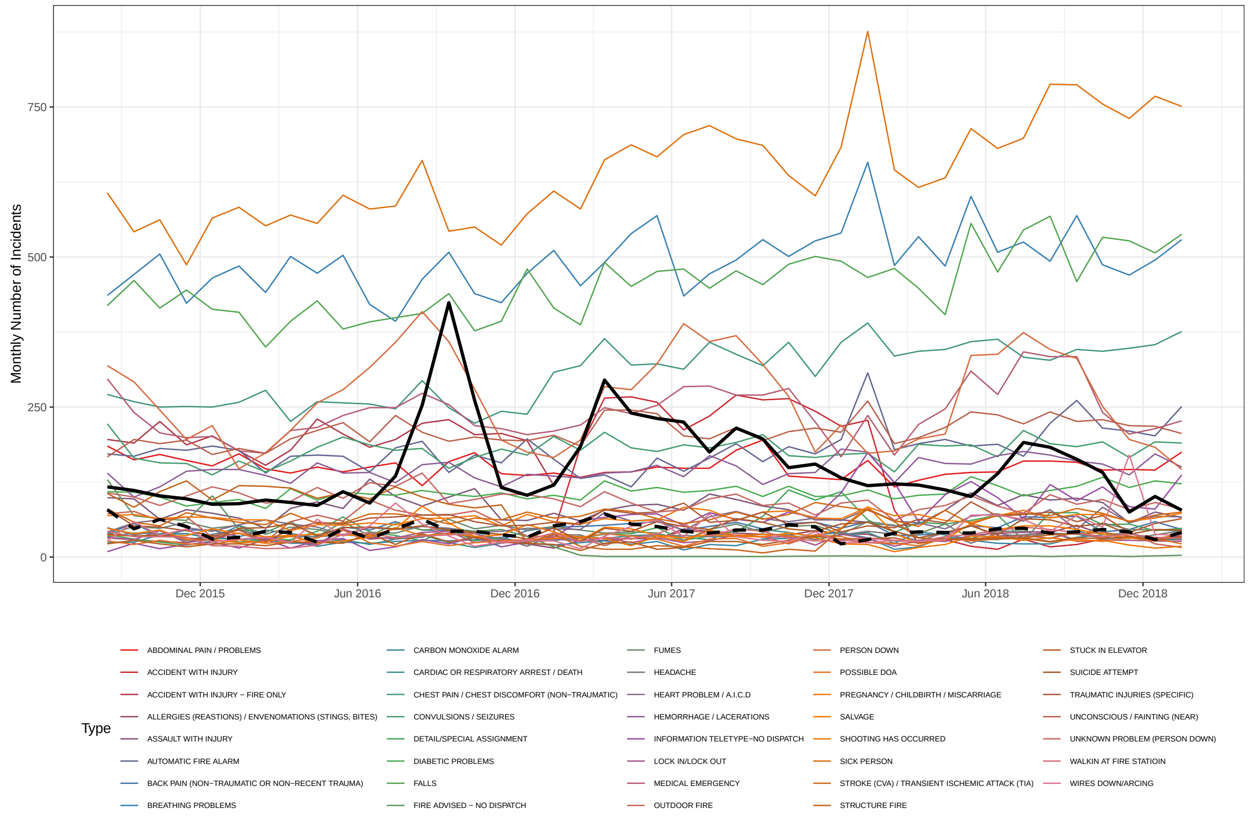

Figure 7: Comparison of monthly counts of EMS calls for different types of incidents. The solid black line shows the number of heroin-related overdose incidents. The dotted black line shows the number of incidents classified as general overdose/poisoning. All incident type groups with more than $1,000$ incidents are included.

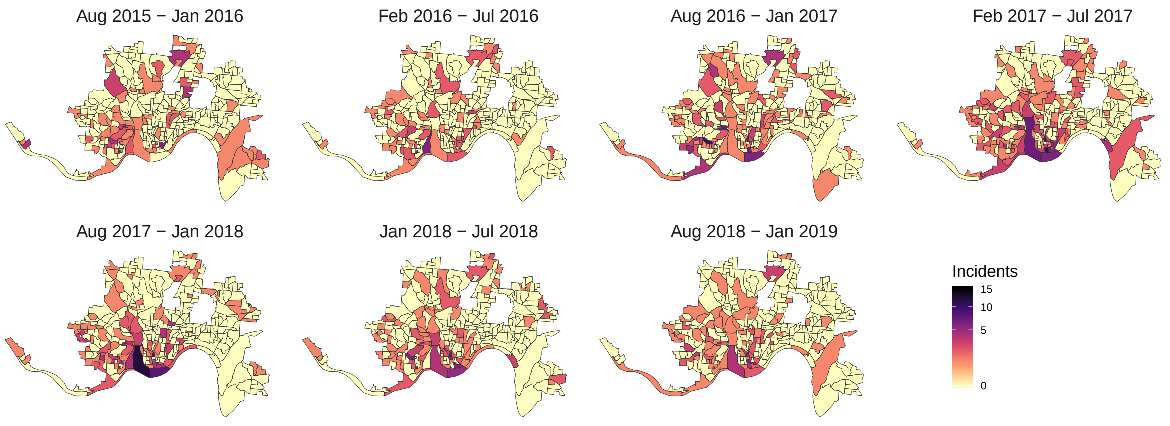

Figure 8: Number of the incidents by block groups and 6-month windows. Geographic boundary files were downloaded from the United States Census, TIGER, Geodatabase (United States Census Bureau, 2017).

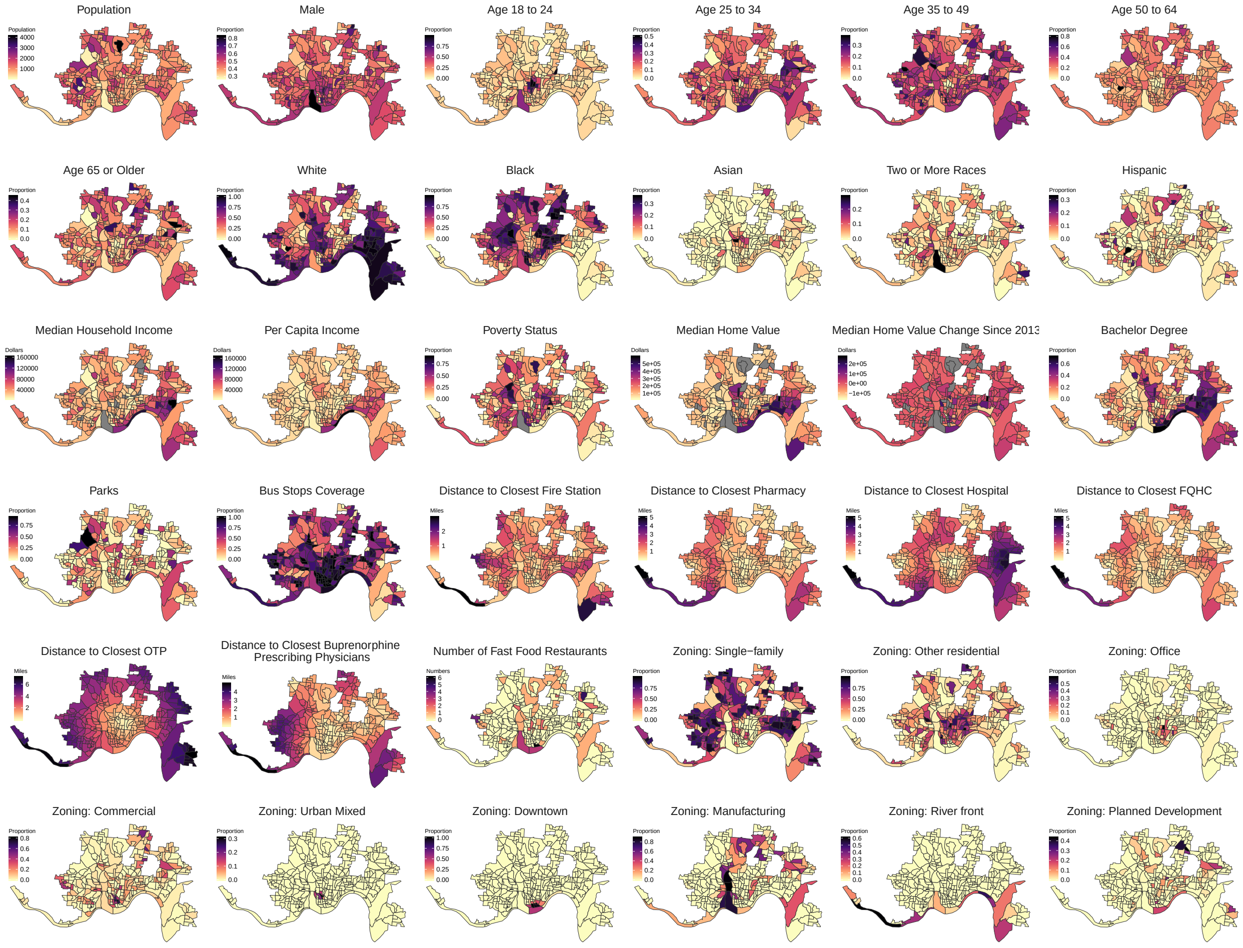

Figure 9: Spatial distribution of time-invariant covariates by block group. Geographic boundary files were downloaded from the United States Census, TIGER, Geodatabase (United States Census Bureau, 2017).

2 Spatial and temporal covariates

Table 1 lists all the covariates used in the model. The age group breakdowns follow from those used in (Bates et al., 2018). Figure 9 shows the spatial distribution of all time-invariant covariates in the analysis.

Covariate	Mean	SD	Source
Log of Population	6.853	0.536	U.S. Census Bureau (2017)
Proportion Male	0.488	0.083	U.S. Census Bureau (2017)
Proportion Aged 18-24	0.126	0.154	U.S. Census Bureau (2017)
Proportion Aged 25-34	0.180	0.092	U.S. Census Bureau (2017)
Proportion Aged 35-49	0.173	0.071	U.S. Census Bureau (2017)
Proportion Aged 50-64	0.187	0.091	U.S. Census Bureau (2017)
Proportion Aged 65 up	0.125	0.083	U.S. Census Bureau (2017)
Proportion White	0.510	0.301	U.S. Census Bureau (2017)
Proportion Black	0.425	0.311	U.S. Census Bureau (2017)
Proportion Asia	0.018	0.038	U.S. Census Bureau (2017)
Proportion Two or More Races	0.034	0.044	U.S. Census Bureau (2017)
Proportion Hispanic	0.035	0.054	U.S. Census Bureau (2017)
Median Household Income ($100K)	0.433	0.255	U.S. Census Bureau (2017)
Per Capita Income ($100K)	0.283	0.196	U.S. Census Bureau (2017)
Proportion in Poverty	0.222	0.203	U.S. Census Bureau (2017)
Median Home Value ($1M)	0.153	0.112	U.S. Census Bureau (2017)
Change in Median Home Value ($1M)	0.007	0.045	U.S. Census Bureau (2017, 2013)
Proportion Bachelor's Degree or Higher	0.229	0.184	U.S. Census Bureau (2017)
Proportion Parks	0.114	0.154	City of Cincinnati (2014)
Proportion 1/4-mile Bus Coverage	0.695	0.260	City of Cincinnati (2014)
Distance to Fire Department (10 miles)	0.783	0.444	City of Cincinnati (2018)
Distance to Pharmacies (10 miles)	0.092	0.075	HRSA Data Warehouse (2019)
Distance to Hospitals (10 miles)	0.177	0.111	HRSA Data Warehouse (2019)
Distance to FQHC (10 miles)	0.083	0.065	HRSA Data Warehouse (2019)
Distance to OTP (10 miles)	0.322	0.167	SAMHSA (2019)
Distance to Buprenorphine Practitioners (10 miles)	0.136	0.111	SAMHSA (2019)
Number of Fast Food Restaurants	0.257	0.717	Cincinnati Bell (2019)
Proportion Single Family Zoning	0.467	0.326	City of Cincinnati (2014)
Proportion Other Residential Zoning	0.227	0.230	City of Cincinnati (2014)
Proportion Office Zoning	0.018	0.059	City of Cincinnati (2014)
Proportion Commercial Zoning	0.079	0.128	City of Cincinnati (2014)
Proportion Urban Mixed Zoning	0.003	0.025	City of Cincinnati (2014)
Proportion Downtown Development Zoning	0.019	0.116	City of Cincinnati (2014)
Proportion Manufacturing Zoning	0.068	0.164	City of Cincinnati (2014)
Proportion Riverfront Zoning	0.011	0.061	City of Cincinnati (2014)
Proportion Planned Development Zoning	0.018	0.054	City of Cincinnati (2014)
Temperature (°F)	55.793	15.988	National Weather Service (2019)
Precipitation (Inch)	4.140	1.668	National Weather Service (2019)
Crime Rate Per Population	0.011	0.014	City of Cincinnati (2019)

Table 1: List of spatial covariates used in this study. Demographic and social economic variables are obtained from the 2013–2017 5-year estimates of the American Community Survey (ACS) (U.S. Census Bureau, 2017). Zoning and other public map are obtained from the Cincinnati Area Geographic Information System (CAGIS) (City of Cincinnati, 2014).

3 Details of the Space-time Smoothing Model

3.1 Hyperprior choices

The priors on the random effects are formulated as the follows,

$$
\begin{aligned}
\alpha_i &= \frac{1}{\tau_\alpha}(\sqrt{\phi_\alpha}u_i + \sqrt{1-\phi_\alpha}v_i), \quad u_i \sim \text{Normal}(0, \boldsymbol{Q}^{-}), \quad v_i \sim \text{Normal}(0, \boldsymbol{I}), \\
\phi_t &= \gamma_t + \eta_t \\
\gamma_t &= \rho_\gamma \gamma_{t-1} + \epsilon_t, \quad \epsilon_t \sim \text{Normal}(0, \frac{1}{\tau_\gamma}\boldsymbol{I}), \\
\eta_t &\sim \text{Normal}(0, \frac{1}{\tau_\eta}\boldsymbol{I}), \\
vec(\delta_{it}) &\sim \text{Normal}(0, \frac{1}{\tau_\delta}\boldsymbol{I}),
\end{aligned}
$$

where $\boldsymbol{Q}^{-}$ is the generalized inverse of the matrix $\boldsymbol{Q}$. The BYM prior on the spatial random effect α_i is parameterized using the proposal introduced in Riebler et al. (2016). In order to facilitate interpretability of hyperprior choices, both the BYM and random walk models are rescaled to have unit generalized marginal variance (Sørbye and Rue, 2014), and we use the penalised complexity (PC) priors (Simpson et al., 2017) for the prior distribution of hyperparameters.

The hyperprior distributions are chosen with the PC framework (Riebler et al., 2016). For the precision parameters, we let $\tau_\alpha, \tau_\gamma, \tau_\eta, \tau_\delta \sim$ PC$(0.2/0.31, 0.01)$, which corresponds to $\Pr(1/\sqrt{\tau} > 0.2/0.31) = 0.01$, and leads to the prior standard deviation of the marginal log relative risk being 0.2. For ϕ_α, we let $\phi_\alpha \sim$ PC$(0.5, 2/3)$, corresponding to $\Pr(\phi_\alpha < 0.5) = 2/3$, which puts more prior weights on the unstructured component of the BYM model. The parameter $\rho_\gamma \sim$ PC$(0.9, 0.9)$, corresponds to $\Pr(\rho < 0.9) = 0.9$.

3.2 Posterior distribution of hyperparameters

Figure 10 shows the posterior distribution of the hyperparameters in the model. The posterior distributions of all standard deviations are centered away from 0, indicating the existence of spatial and temporal variations. The posterior distributions of ρ and ϕ are centered at non-zero values, indicating evidence of structured correlations in the residuals.

3.3 Model selection

In the analysis, we included the log population as a covariate in the model. Here we also consider the population as the offset, or exposure, in the Poisson regression, i.e., $y_{it} \sim$ Poisson$(N_i\lambda_{it})$. We compared the two parameterization under the following models:

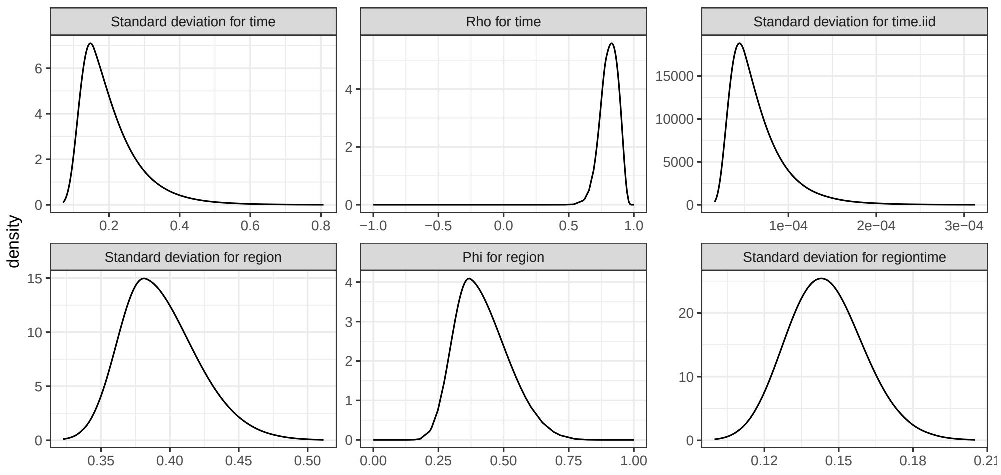

Figure 10: Posterior marginal distributions of the hyperparameters.

$$\begin{aligned}
\text{Fixed effects only:} \quad & \log(\lambda_{it}) = \boldsymbol{x}_{it}^T\boldsymbol{\beta} \\
\text{Fixed and independent random effects:} \quad & \log(\lambda_{it}) = \boldsymbol{x}_{it}^T\boldsymbol{\beta} + \delta_{it} \\
\text{Fixed, temporal, and independent random effects:} \quad & \log(\lambda_{it}) = \boldsymbol{x}_{it}^T\boldsymbol{\beta} + \gamma_t + \eta_t + \delta_{it} \\
\text{Full space-time smoothing model:} \quad & \log(\lambda_{it}) = \boldsymbol{x}_{it}^T\boldsymbol{\beta} + \alpha_i + \gamma_t + \eta_t + \delta_{it}
\end{aligned}$$

In order to determine whether our full model is required to adequately describe variability in the data or if some features can be simplified, we compare the different models using DIC (Spiegelhalter et al., 2002) and Watanabe Akaike Information Criterion (WAIC) (Watanabe, 2010). DIC and WAIC identify the most appropriate model among candidate models based on both model fit and complexity, with smaller values of the metric being preferred. The results are summarized in Table 2. The estimated effective number of parameters in the proposed space-time model is much smaller than simple random effect model due to its ability to borrow information across observations. Modeling log population as a covariate yields slightly better DIC and WAIC, and improved interpretability of the coefficients.

To further examine the differences between the count model presented in the main paper and the risk model that include population as offset, we include the result from the latter model in this subsection. Figure 11 shows the summary of the posterior distributions of the fixed effects in the model. Almost all coefficients with 95% posterior credible interval not containing or almost not containing 0 are similar to the results in the main paper. The only exception is the coefficients associated with proportion of residents aged $50-64$ years old, which has a stronger association with the risk of heroin-related overdose than the counts. Figure 12 shows the posterior distributions of the spatial and temporal random effects and are again similar to the results presented in the main paper. However, one block group, Queensgate (the dark red region at the south end of the city in Figure 12), which has only 196 residents, is estimated to have a much higher spatial random effect due to its high number of incidents per resident.

In general, any misspecification of the exposure, i.e., the population estimates from American Com-

Population	model	DIC (N_{eff})	WAIC (N_{eff})
As offset	Fixed effects only	20901.266 (39)	20937.857 (73)
	Fixed and independent random effects	19277.081 (2015.2)	19328.699 (1682.6)
	Fixed, temporal, and independent random effects	18989.321 (1697.4)	19065.625 (1469.8)
	Full space-time smoothing model	**18242.396 (949.3)**	**18301.036 (887.4)**
As covariate	Fixed effects only	20732.545 (40)	20762.056 (68.2)
	Fixed and independent random effects	19247.408 (1948)	19293.57 (1628)
	Fixed, temporal, and independent random effects	18949.287 (1611)	19020.276 (1400.4)
	Full space-time smoothing model	**18239.461 (947.3)**	**18296.513 (884.3)**

Table 2: Comparison of the model fit using DIC and WAIC. N_{eff} is the effective number of parameters in each model. The best-fitting model within each class of models is highlighted in bold. Modeling log population as a covariate yields slightly better DIC and WAIC.

munity Survey, can make the interpretation of regression coefficients and random effects as risks of heroin-related overdose problematic. Therefore, considering the better model fit evaluation in Table 2 and the similar directions of coefficients in both models, we recommend the count model presented in the main paper.

3.4 Predictive performance

The space-time model can also be used for prediction. Figure 13 shows the comparison of the predicted and actual counts of incidents for the last 10 months using the model fitted on the subset of the data before April, 2018. The observed counts and predictions from the fitted model exhibit a close correspondence.

3.5 Sensitivity to jittered GPS location

We evaluate the impact of random jittering of the released EMS data on estimated model coefficients. The Office of Performance and Data Analytics offsets the original coordinate values to within approximately 100 yards (300 feet) in any direction (City of Cincinnati, Office of Performance & Data Analytics, 2019). Since the original location of each incident is unknown, we imputed latent incident GPS locations given the reported locations by randomly jittering the location of each incident in the same way. We then fit the model using the generated dataset with the new locations, and repeated these steps 100 times. Figure 14 compares the estimated fixed effects in the main analysis to the parameters estimated from these 100 datasets with additional jittering. Figure 15 shows the estimated spatial random effects under the same comparison. Variations in the fixed effects and spatial random effects due to additional jittering of the incident locations are relatively small and show no significant changes to the conclusions in the main analysis. Figure 16 shows the estimated temporal random effects, which do not change when the GPS locations of incidents are changed. This analysis shows that the potential impact of jittering on estimated model parameters is small.

Fixed effect	exp(Mean)	95% Posterior CI
**Proportion Male**	**5.369**	**(1.594, 18.142)**
Proportion Aged 18−24	0.385	(0.106, 1.398)
Proportion Aged 25−34	4.503	(0.741, 27.469)
**Proportion Aged 35−49**	**19.204**	**(3.216, 114.958)**
**Proportion Aged 50−64**	**8.503**	**(2.015, 35.579)**
Proportion Aged 65 up	3.544	(0.717, 17.639)
Proportion White	0.264	(0.010, 7.301)
Proportion Black	0.110	(0.004, 3.061)
Proportion Asia	0.130	(0.002, 9.417)
Proportion Two or More Races	5.574	(0.103, 306.535)
Proportion Hispanic	1.514	(0.234, 9.750)
**Median Household Income ($100K)**	**0.450**	**(0.227, 0.887)**
Per Capita Income ($100K)	1.620	(0.570, 4.573)
Proportion in Poverty	1.173	(0.609, 2.266)
Median Home Value ($1M)	0.439	(0.110, 1.744)
Change in Median Home Value ($1M)	0.382	(0.034, 4.189)
**Proportion Bachelor's Degree or Higher**	**0.123**	**(0.025, 0.605)**
**Proportion Parks**	**2.591**	**(1.199, 5.602)**
Proportion 1/4−mile Bus Coverage	1.171	(0.740, 1.854)
Distance to Fire Departments (10 miles)	0.777	(0.597, 1.012)
**Distance to Pharmacies (10 miles)**	**24.547**	**(1.615, 342.094)**
Distance to Hospitals (10 miles)	0.845	(0.703, 1.012)
Distance to FQHC (10 miles)	0.191	(0.013, 2.921)
**Distance to OTP (10 miles)**	**0.138**	**(0.035, 0.546)**
Distance to Buprenorphine Practitioners (10 miles)	1.762	(0.200, 14.318)
**Number of Fast Food Restaurants**	**1.169**	**(1.032, 1.322)**
Proportion Single Family Zoning	1.148	(0.540, 2.441)
Proportion Other Residential Zoning	0.924	(0.378, 2.259)
Proportion Office Zoning	1.459	(0.245, 8.619)
**Proportion Commercial Zoning**	**15.600**	**(5.095, 47.981)**
Proportion Urban Mixed Zoning	13.074	(0.390, 435.566)
**Proportion Downtown Development Zoning**	**12.808**	**(3.990, 41.087)**
**Proportion Manufacturing Zoning**	**5.677**	**(2.214, 14.600)**
**Proportion Riverfront Zoning**	**10.983**	**(1.818, 66.846)**
Proportion Planned Development Zoning	0.979	(0.170, 5.607)
**Temperature (F)**	**1.011**	**(1.002, 1.020)**
Precipitation (Inch)	0.983	(0.942, 1.024)
Crime Rate Per Population	1.448	(0.112, 17.576)

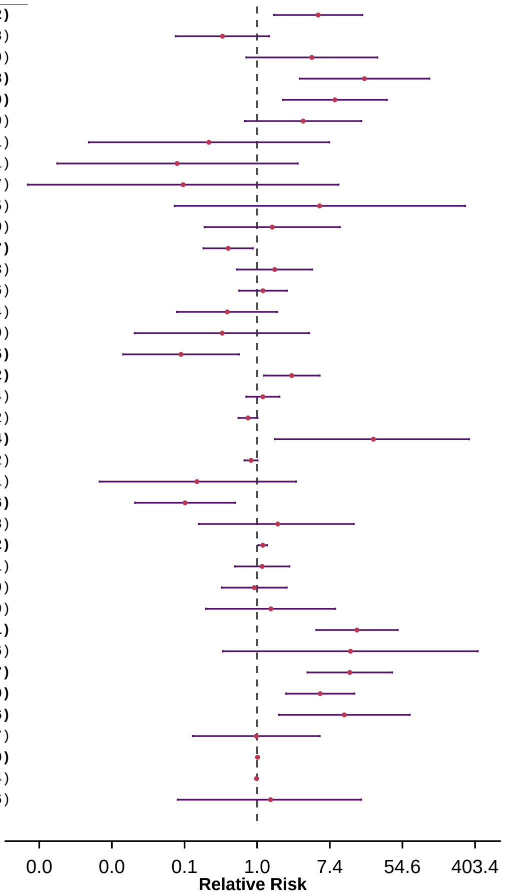

Figure 11: Summary of the posterior means and 95% credible intervals of the coefficients for the fixed effects **in the risk model**. The regression coefficients are exponentiated to represent relative risks in the table.

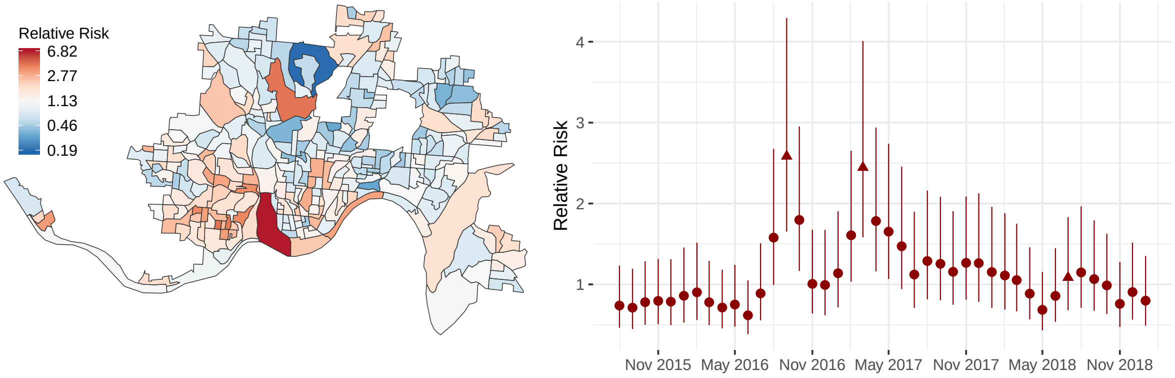

Figure 12: Posterior means of the autoregressive spatial (α_i) and temporal (ϕ_t) effects **in the risk model**. The random effects are exponentiated to represent relative risks. Larger values correspond to higher log counts of incidents. The error bars indicate 95% posterior credible intervals. The triangle dots correspond to the three major peaks in overdose incidents at September 2016, March 2017, and July 2018. Geographic boundary files were downloaded from the United States Census, TIGER, Geodatabase (United States Census Bureau, 2017).

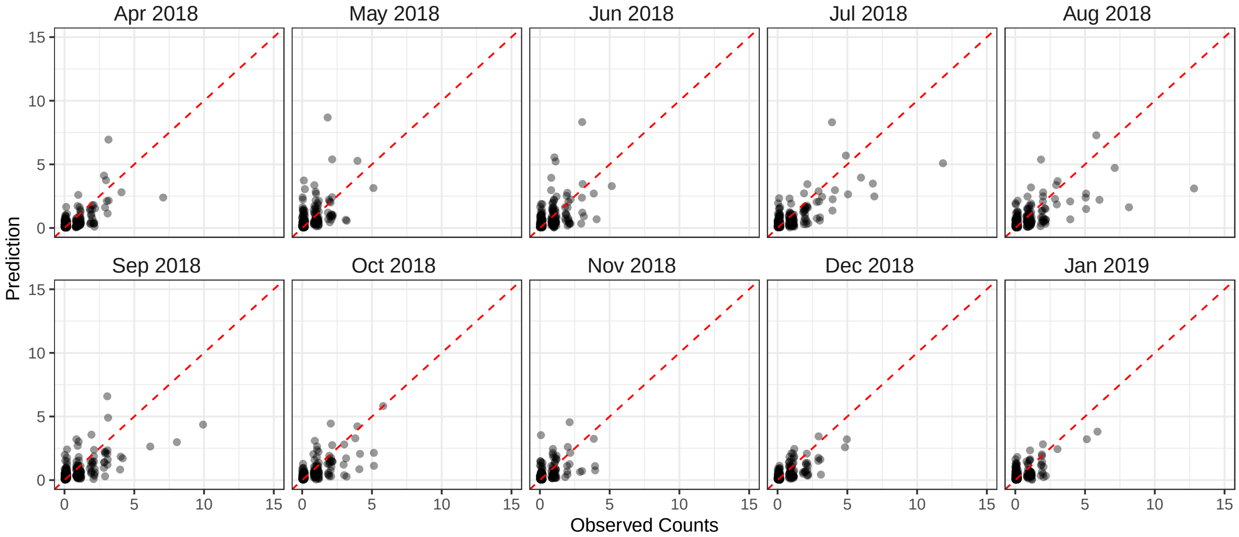

Figure 13: Comparison of predicted and the observed counts of incidents for the last 10 months in the dataset. The predicted counts are posterior means fitted on the subset of the data before April, 2018. The observed counts are slightly horizontally jittered to reduce overlap.

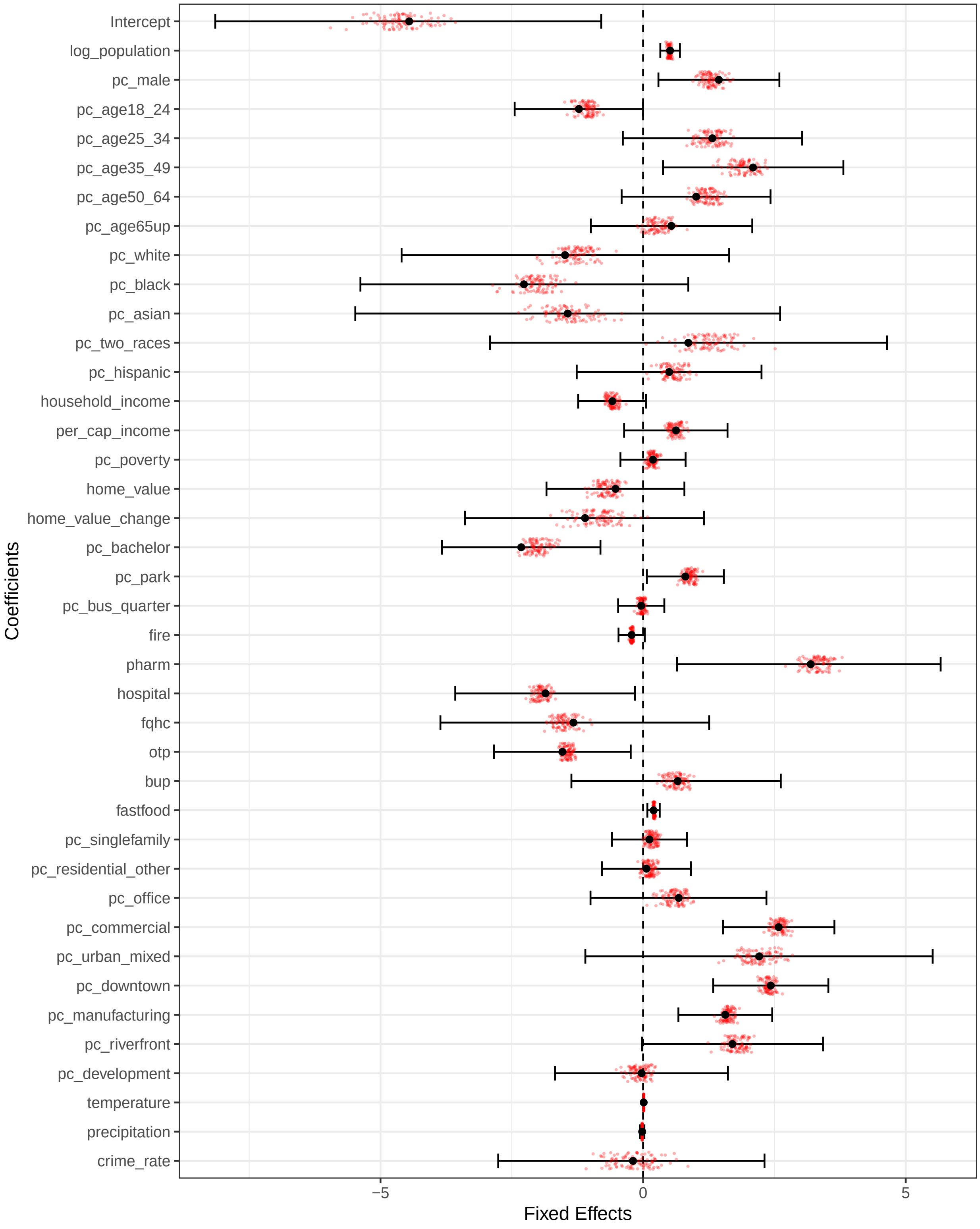

Figure 14: Comparison of posterior means and 95% credible intervals of the fixed effects (black dot and error bars) estimated from the actual EMS data with the posterior means of the fixed effects estimated from 100 datasets with additional jittering (red dots).

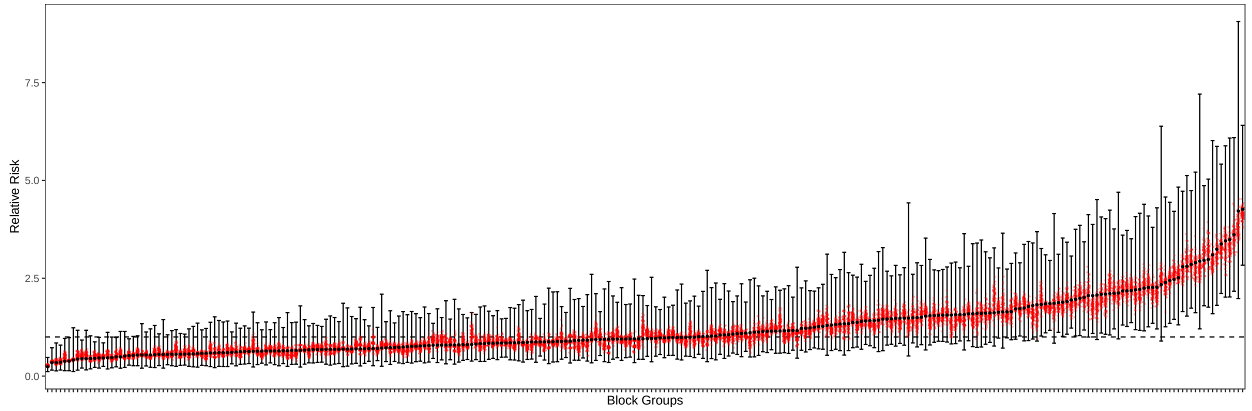

Figure 15: Comparison of posterior means and 95% credible intervals of the spatial random effects (black dot and error bars) estimated from the actual EMS data with the posterior means of the spatial random effects estimated from 100 datasets with additional jittering (red dots). The block groups are ordered by the posterior means of the spatial random effects.

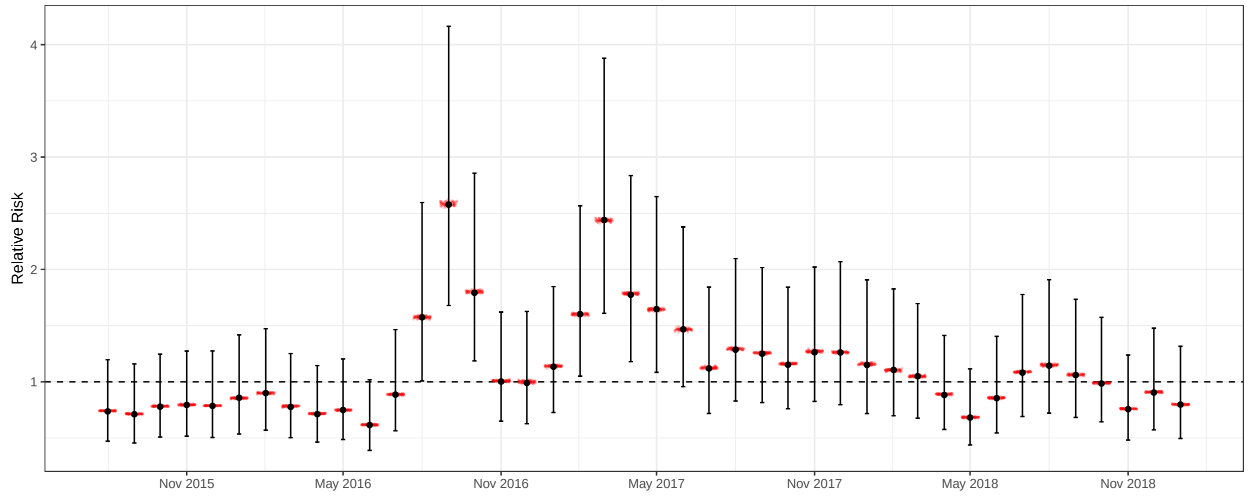

Figure 16: Comparison of the posterior means and 95% credible intervals of the temporal random effects (black dot and error bars) estimated from the actual EMS data with the posterior means of the temporal random effects estimated from 100 datasets with additional jittering (red dots).

3.6 Sensitivity to incident type classification

We evaluated the impact of potential misclassification of incident types on parameter estimates in three sensitivity analyses. We examined the main results presented in the paper using heroin-related overdose incidents, with the same regression analysis for the following outcomes:

1. We fit the space-time regression model on all overdose incidents, i.e., both heroin-related overdose, and the general category of overdose, ‘overdose/poisoning (ingestion)’, instead of just the incidents related to heroin.
2. We fit the space-time regression model on the overdoses that are not heroin-related, using all overdose incidents as an offset in the model.
3. We fit the space-time regression model on incidents labeled “pregnancy/childbirths/miscarriage”.

The first analysis allows us to evaluate the sensitivity of our main results under potential misclassification of overdose types. Figure 17 shows parameter estimates under the assumption that overdoses that were not classified as involving heroin at the time of dispatch were actually all heroin-related. This analysis shows that changes in parameter estimates are minimal even if all incidents labeled as not heroin-related were misspecified. Figures 18 and 19 show similar comparisons of the spatial and temporal random effects respectively. These results also show little difference between the posterior summaries of these random effects regardless of whether the overdose incidents are related to heroin were misclassified. This analysis suggests that our main results are robust to potential misclassification of the nature of overdose.

The second analysis evaluates whether the rate of overdose incidents being labeled as heroin-related or not is different depending on covariates, time, or location. Figure 20 shows that the 95% posterior credible intervals of most of the fixed effects do not cover 0 (or equivalently, the CI of relative risk does not cover 1). Figure 21 also shows that the 95% posterior credible intervals of almost all temporal random effects and all spatial random effects cover 0 as well. This indicates that classification of overdose incidents as not related to heroin does not have strong spatial or temporal patterns, nor strongly related to covariates. That is, our main analysis is unlikely to contain bias due to overdose being systematically misclassified as related to heroin in specific locations or times.

The last analysis is suggested by a reviewer. It evaluates whether our analysis reflects a generic pattern of EMS calls that are common to other types of incidents, rather than patterns specific to heroin-related overdose. Figure 22 compares the fixed effects in the main analysis to the same models fitted on incidents classified as “pregnancy/childbirths/miscarriage”, which is unlikely to share the same association to the covariates as heroin-related overdose. This analysis shows that the two types of incidents give very different estimates of fixed effects. Figures 23 and 24 compare posterior summaries of the spatial and temporal random effects respectively. Figure 23 shows that the unadjusted spatial heterogeneity patterns in the two outcomes are very different, and Figure 24 shows that there is much more temporal heterogeneity in the analysis of heroin-related overdose than pregnancy. This analysis confirms that the relationship to covariates and patterns in the estimated spatial and temporal distributions found in heroin-related incidents is not common to all types of incidents.

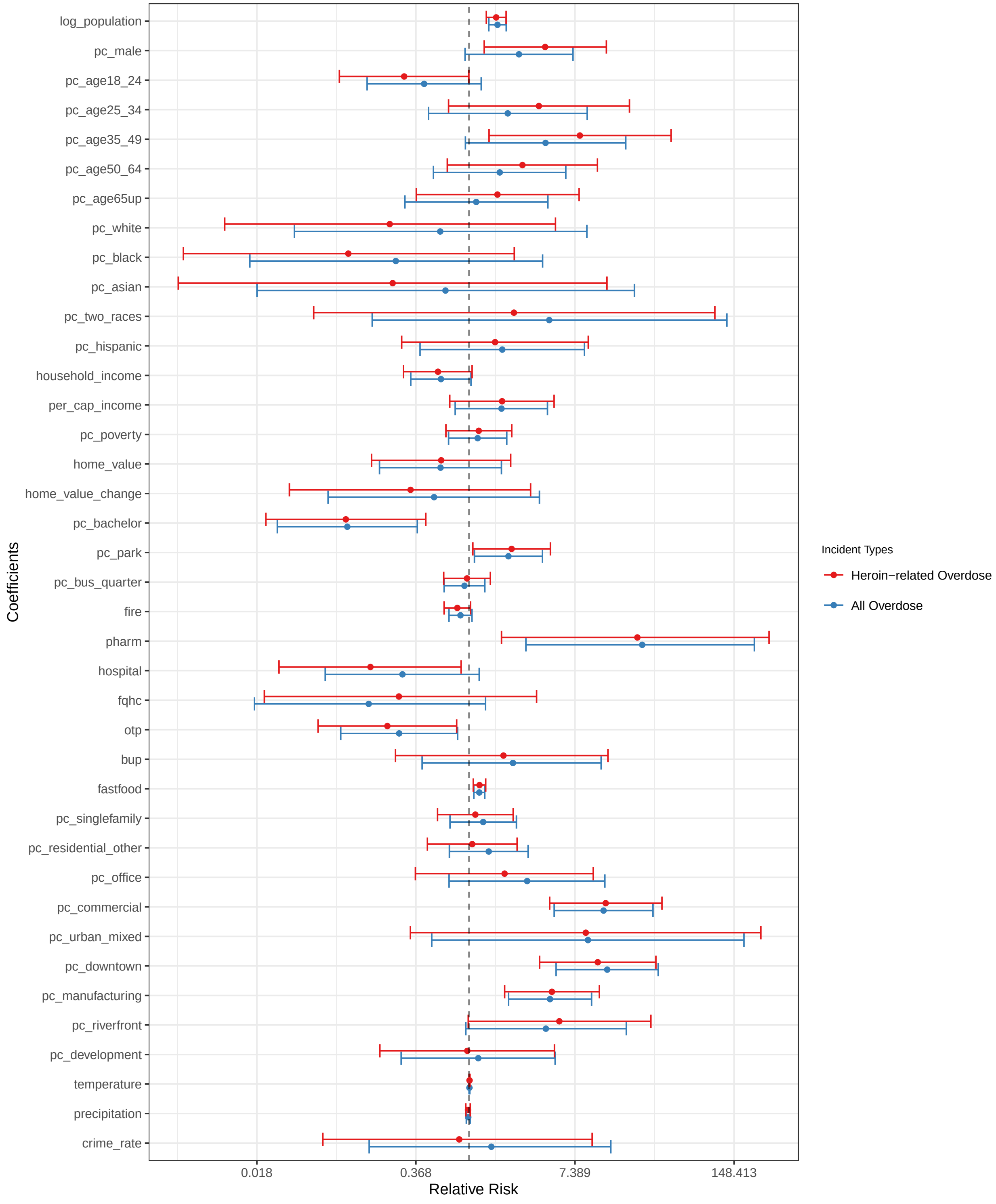

Figure 17: Comparison of posterior means and 95% credible intervals of the fixed effects estimated using only heroin-related overdose incidents and using all overdose incidents.

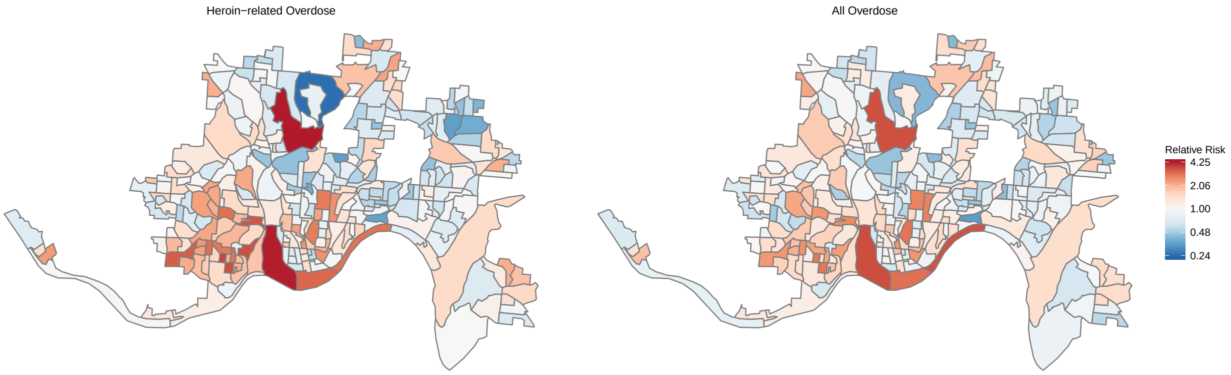

Figure 18: Comparison of posterior means of the spatial effects estimated using only heroin-related overdose incidents and using all overdose incidents. Geographic boundary files were downloaded from the United States Census, TIGER, Geodatabase (United States Census Bureau, 2017).

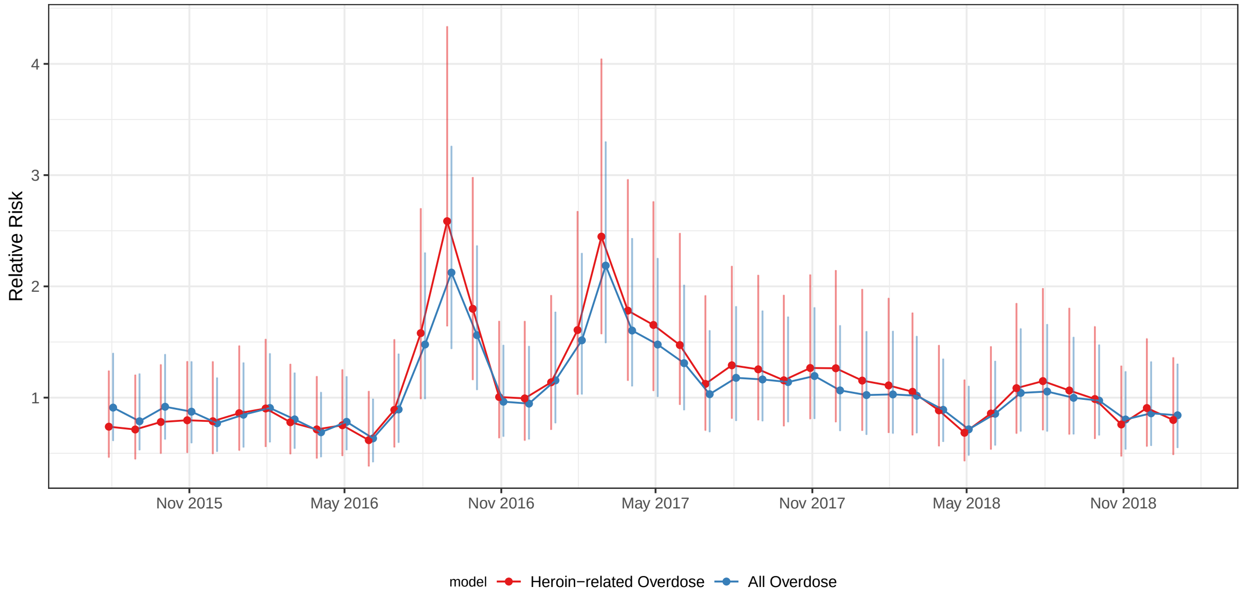

Figure 19: Comparison of posterior means and 95% credible intervals of the temporal random effects estimated using only heroin-related overdose incidents and using all overdose incidents.

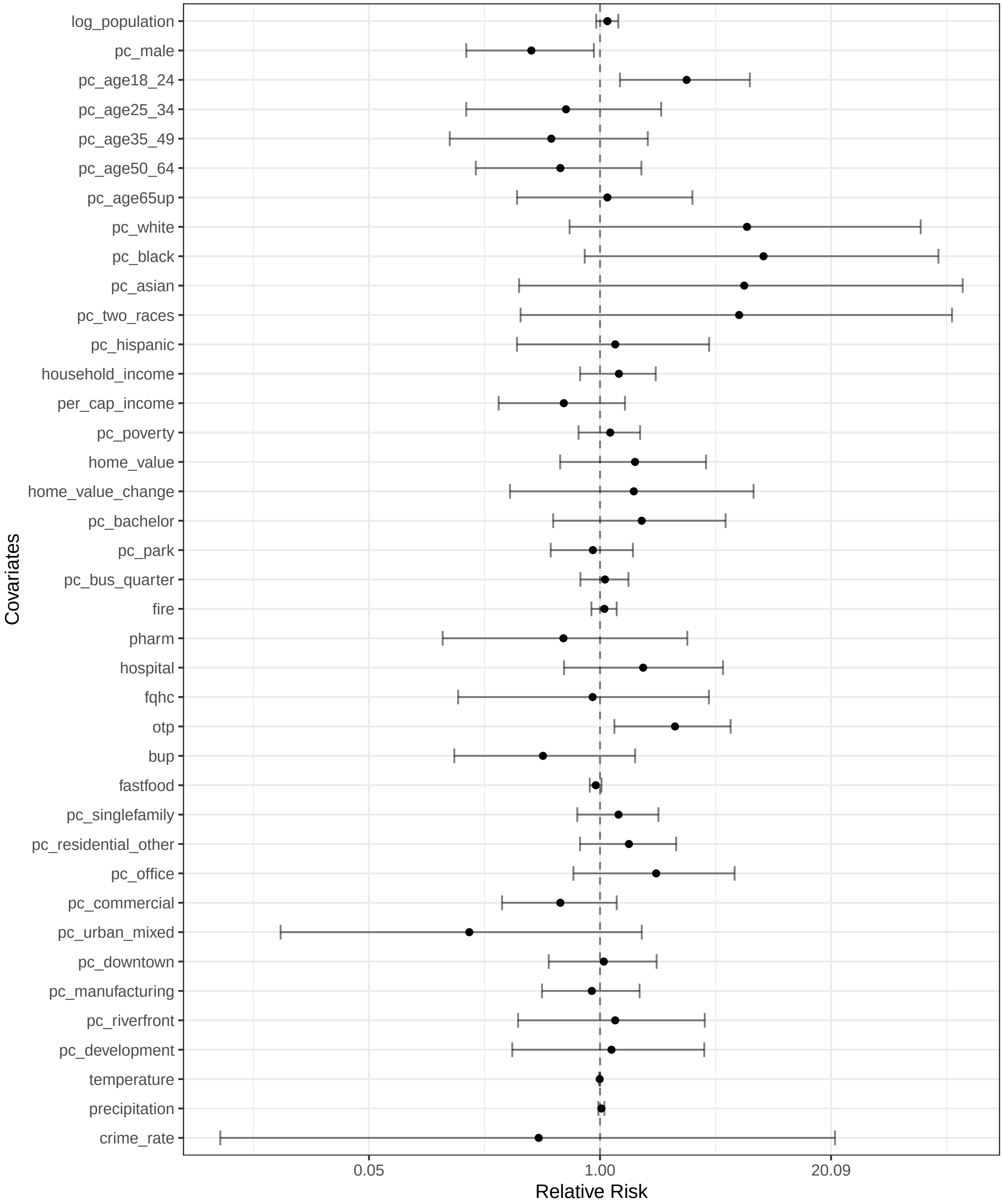

Figure 20: Posterior means and 95% credible intervals of the fixed effects estimated using only non heroin-related overdose incidents with the number of overdose incidents as the offset in the model.

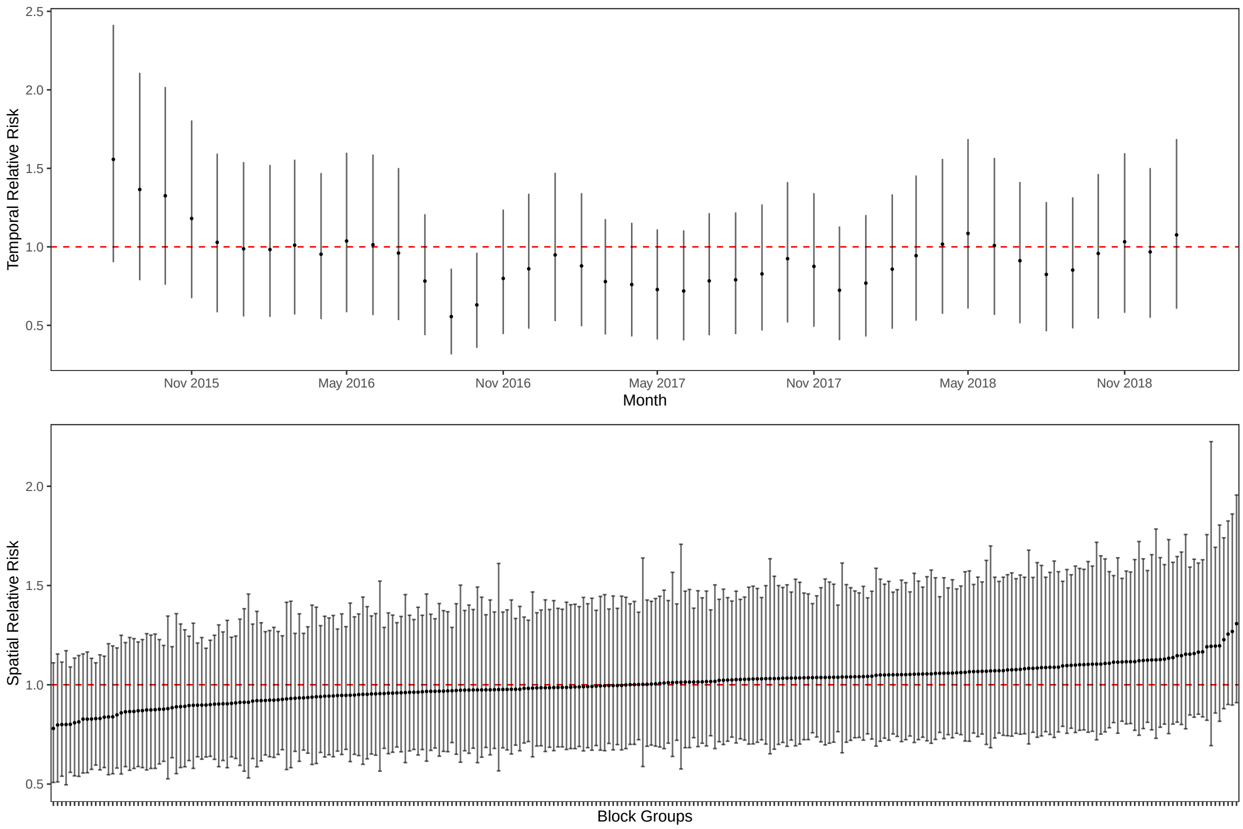

Figure 21: Posterior means and 95% credible intervals of the temporal (upper panel) and spatial (lower panel) effects estimated using only non heroin-related overdose incidents and using all overdose incidents as the offset in the model. The block groups are arranged in the order of the posterior means of the spatial random effects.

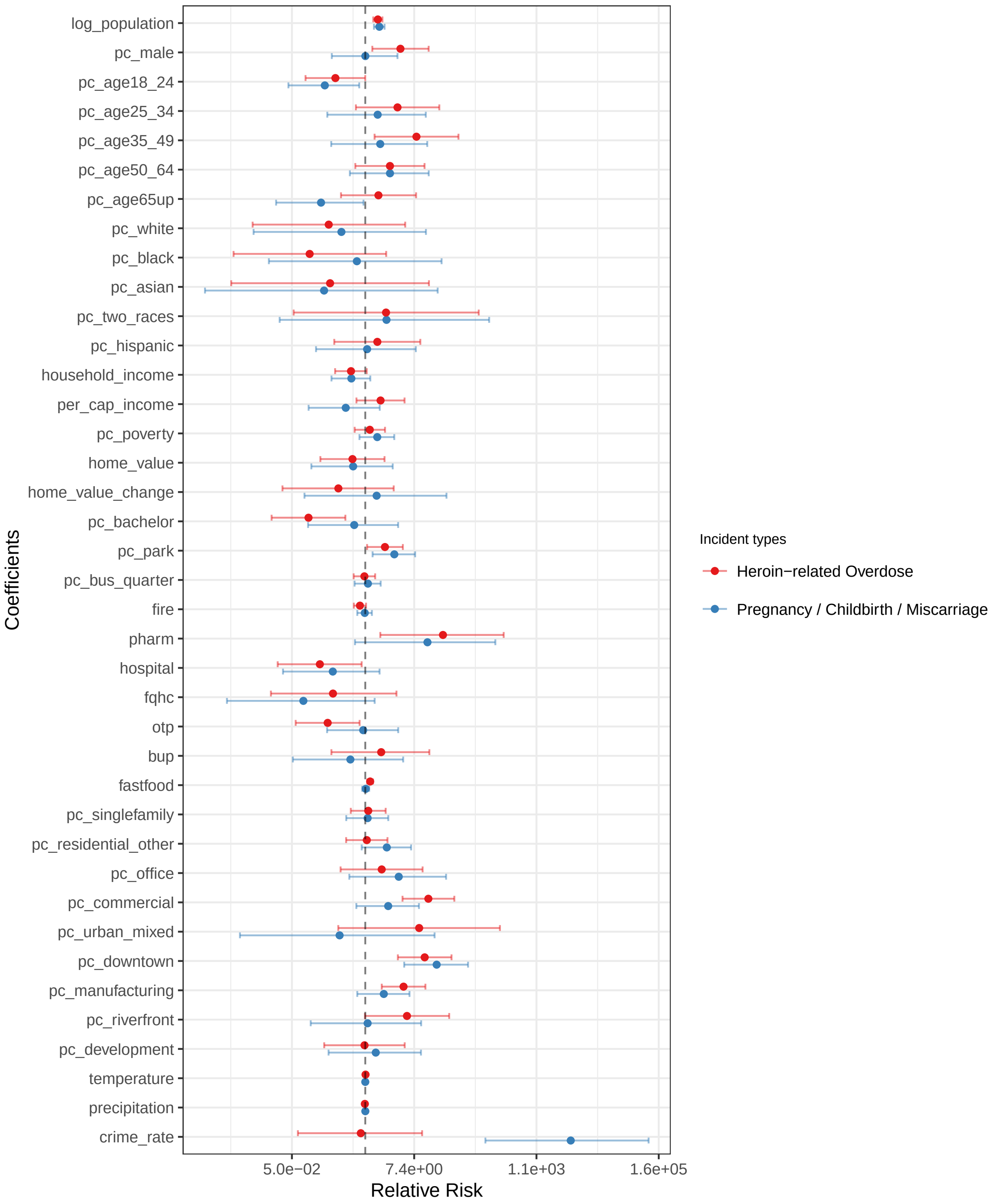

Figure 22: Comparison of posterior means and 95% credible intervals of the fixed effects estimated using heroin-related overdose incidents and using incidents corresponding to pregnancy, childbirth, or miscarriage.

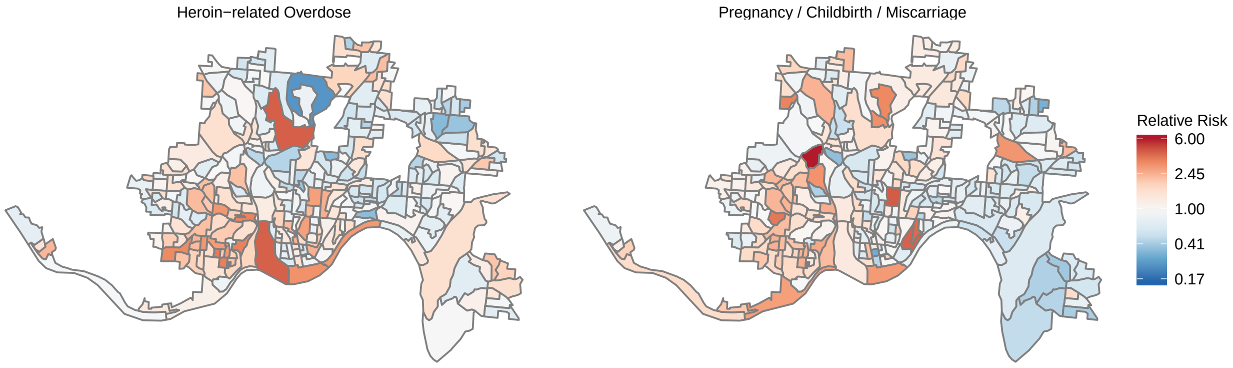

Figure 23: Comparison of posterior means of the spatial effects estimated using heroin-related overdose incidents and using incidents corresponding to pregnancy, childbirth, or miscarriage. Geographic boundary files were downloaded from the United States Census, TIGER, Geodatabase (United States Census Bureau, 2017).

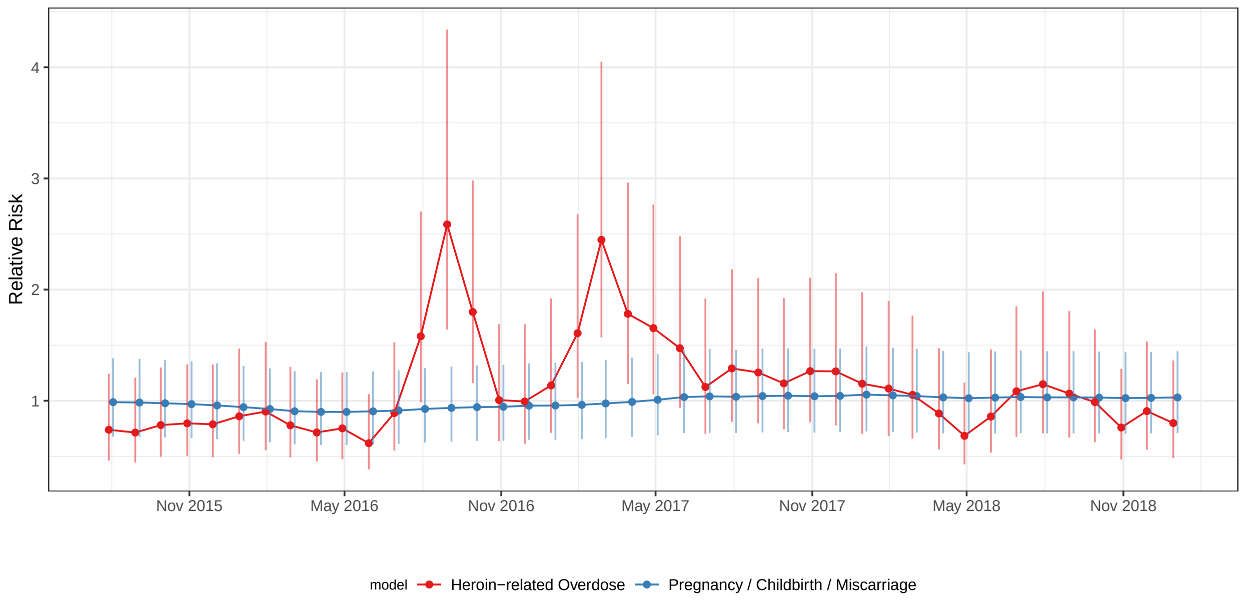

Figure 24: Comparison of posterior means and 95% credible intervals of the temporal random effects estimated using heroin-related overdose incidents and using incidents corresponding to pregnancy, childbirth, or miscarriage.

3.7 Seasonality

Figure 25 shows the posterior marginal distributions of the temporal random effects, summarized by calendar month. There is no clear pattern of seasonality or systematic differences in the marginal distributions of temporal effects when aggregated by month.

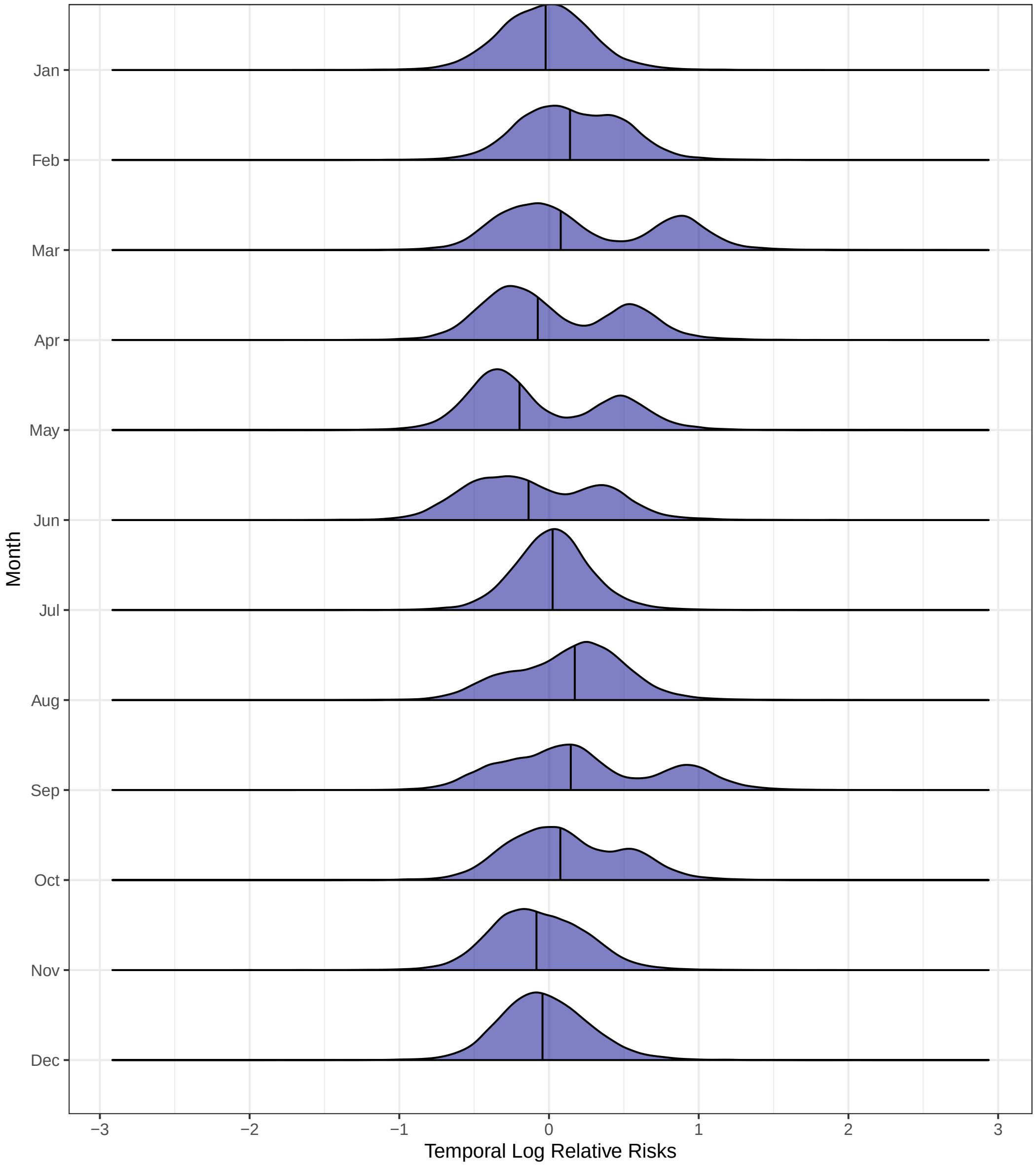

Figure 25: Comparison of posterior marginal distributions of temporal random effects, by calendar month.

References

Bates, S., Leonenko, V., Rineer, J., and Bobashev, G. (2018). Using synthetic populations to understand geospatial patterns in opioid related overdose and predicted opioid misuse. *Computational and Mathematical Organization Theory*, pages 1–12.

Cincinnati Bell (2019). White Pages. Retrieved from: https://www.cincinnatibell.com/whitepages; Accessed Jan 10, 2019.

City of Cincinnati (2010). Cincinnati Area Geographic Information System. Available from: https://data-cagisportal.opendata.arcgis.com/datasets/572561553c9e4d618d2d7939c5261d46_0; Accessed Feb 1, 2019.

City of Cincinnati (2014). Cincinnati Area Geographic Information System. Available from: http://cagismaps.hamilton-co.org/cagisportal/mapdata/; Accessed Feb 1, 2019.

City of Cincinnati (2018). CFD Fire Districts. Available from: https://www.cincinnati-oh.gov/fire/about-fire/cfd-fire-districts/; Accessed Nov 15, 2018.

City of Cincinnati (2019). PDI (Police Data Initiative) Crime Incidents. Data retrieved from Open Data Cincinnati, https://data.cincinnati-oh.gov/Safer-Streets/PDI-Police-Data-Initiative-Crime-Incidents/k59e-2pvf; Accessed Feb 1, 2019.

City of Cincinnati, Office of Performance & Data Analytics (2019). Personal Communication Aug 9, 2019.

HRSA Data Warehouse (2019). Find a Health Center. Retrieved from: https://findahealthcenter.hrsa.gov; Accessed Dec 6, 2018.

National Weather Service (2019). NOWData - NOAA Online Weather Data. Retrieved from: https://w2.weather.gov/climate/xmacis.php?wfo=iln; Accessed Feb 1, 2019.

Riebler, A., Sørbye, S. H., Simpson, D., and Rue, H. (2016). An intuitive Bayesian spatial model for disease mapping that accounts for scaling. *Statistical Methods in Medical Research*, 25(4):1145–1165.

SAMHSA (2019). OTP Directory. Retrieved from: https://dpt2.samhsa.gov/treatment/directory.aspx; Accessed Nov 30, 2018.

Simpson, D., Rue, H., Riebler, A., Martins, T. G., and Sørbye, S. H. (2017). Penalising model component complexity: A principled, practical approach to constructing priors. *Statistical Science*, 32(1):1–28.

Sørbye, S. H. and Rue, H. (2014). Scaling intrinsic Gaussian Markov random field priors in spatial modelling. *Spatial Statistics*, 8:39–51.

Spiegelhalter, D. J., Best, N. G., Carlin, B. P., and Van Der Linde, A. (2002). Bayesian measures of model complexity and fit. *Journal of the Royal Statistical Society: Series B*, 64(4):583–639.

United States Census Bureau (2017). 2017 cartographic boundary file, current block group for Ohio, 1:500,000. Data retrieved from https://www2.census.gov/geo/tiger/GENZ2017/shp/cb_2017_39_bg_500k.zip; Accessed Jan 31, 2019.

U.S. Census Bureau (2013). 2013 American Community Survey (ACS). Available from: https://factfinder.census.gov/; Accessed Feb 1, 2019.

U.S. Census Bureau (2017). 2017 American Community Survey (ACS). Available from: https://factfinder.census.gov/; Accessed Feb 1, 2019.

Watanabe, S. (2010). Asymptotic equivalence of bayes cross validation and widely applicable information criterion in singular learning theory. *Journal of Machine Learning Research*, 11(Dec):3571–3594.
